# Toward a Comprehensive Map of the Effectors of Rab GTPases

**DOI:** 10.1016/j.devcel.2014.10.007

**Published:** 2014-11-10

**Authors:** Alison K. Gillingham, Rita Sinka, Isabel L. Torres, Kathryn S. Lilley, Sean Munro

**Affiliations:** 1 MRC Laboratory of Molecular Biology, Francis Crick Avenue, Cambridge CB2 0QH, UK; 2 Cambridge Centre for Proteomics, Department of Biochemistry, University of Cambridge, Cambridge CB2 1QR, UK

## Abstract

The Rab GTPases recruit peripheral membrane proteins to intracellular organelles. These Rab effectors typically mediate the motility of organelles and vesicles and contribute to the specificity of membrane traffic. However, for many Rabs, few, if any, effectors have been identified; hence, their role remains unclear. To identify Rab effectors, we used a comprehensive set of *Drosophila* Rabs for affinity chromatography followed by mass spectrometry to identify the proteins bound to each Rab. For many Rabs, this revealed specific interactions with *Drosophila* orthologs of known effectors. In addition, we found numerous Rab-specific interactions with known components of membrane traffic as well as with diverse proteins not previously linked to organelles or having no known function. We confirm over 25 interactions for Rab2, Rab4, Rab5, Rab6, Rab7, Rab9, Rab18, Rab19, Rab30, and Rab39. These include tethering complexes, coiled-coiled proteins, motor linkers, Rab regulators, and several proteins linked to human disease.

## Highlights

• Proteomic screen identifies effectors of *Drosophila* Rabs with a human ortholog
• Specific hits include orthologs of numerous known effectors of mammalian Rabs
• Validated effectors include traffic proteins and those of unknown function
• Orthologs of disease genes CLEC16A, LRRK2, and SPG20 are validated as effectors

## Graphical Abstract

**Figure:**
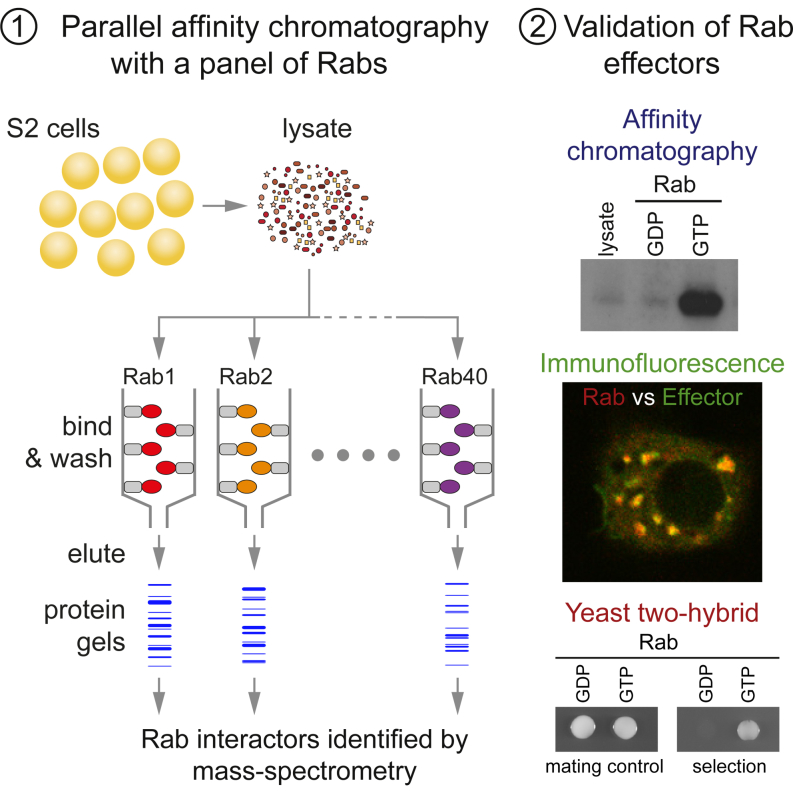

## Teaser

Rab GTPases organize cellular compartments by recruiting specific effectors to organelle membranes. This paper describes affinity chromatography using all *Drosophila* Rabs with a mammalian ortholog. The Rab interactors found include known effectors, tethering complexes, coiled-coil proteins, motor proteins, proteins of unknown function, and several proteins linked to human disease.

## Introduction

The functioning of intracellular compartments requires numerous soluble proteins to be recruited from the cytosol to specific organelles or vesicles. This recruitment is mediated by binding to labile “landmarks,” which are generated only on specific membranes. The principal landmarks used in eukaryotes are the phosophoinositide lipids and the small GTPases of the Arf and Rab families (Di Paolo and De Camilli, 2006, Gillingham and Munro, 2007, Pfeffer, 2013). Of these three families, the largest is the Rabs of which at least twenty were apparently present in the last common ancestor of all eukaryotes (Klöpper et al., 2012). This expanded to over 30 families in early metazoan evolution, and later genome duplications in vertebrates generated closely related paralogs to bring the total in humans to 66. The importance of Rabs is also reflected in mutations in several being linked to disease and by their being modified by intracellular pathogens to subvert membrane traffic (Hutagalung and Novick, 2011, Stenmark, 2009).

Rabs function by cycling between an inactive guanosine diphosphate (GDP)-bound state and an active guanosine triphosphate (GTP)-bound state that is stably associated with membranes as it cannot be recognized for extraction by the Rab chaperone GDP-displacement inhibitor (GDI). Rab effector proteins bind only to the GTP-bound state and so are recruited to only those membranes on which the Rab is active. The activation of Rabs by exchange of GDP for GTP is directed by specific guanine nucleotide exchange factors (GEFs), with the Rabs being then inactivated by specific GTPase activator proteins (GAPs). The mechanisms by which the GEFs and GAPs act together to control the distribution of Rabs is only starting to emerge, but both targeting and local activation seem likely to be important (Barr, 2013, Palamidessi et al., 2013). Most GEFs and GAPs are peripheral membrane proteins and, in some cases, have been found to be recruited by other Rabs, raising the possibility that a network of negative and positive interactions between Rabs sets up the spatial organization of subcellular compartments (Hutagalung and Novick, 2011).

The investigation of the function of individual Rabs has been driven by the identification of the effectors that they recruit to membranes. Most effectors have been identified by yeast-two hybrid screens or affinity chromatography of cytosol using forms of the Rabs that carry mutations that lock them in a GTP-bound state. Known effectors include tethering factors that link organelles and vesicles prior to homotypic or heterotypic fusion, linkers for the motor proteins that direct organelle movement, and regulators of Rabs and other GTPases or lipid species (Hutagalung and Novick, 2011, Pfeffer, 2013). Thus, a picture has emerged of Rabs directing membrane traffic but also serving as landmarks for other proteins that need to reside on a particular compartment or vesicle. However, for many Rabs, there are few, if any, effectors known, and indeed the function of many Rabs remains unclear, including several of those conserved since the emergence of eukaryotes.

Thus, the identification of more Rab effectors could reveal much about the organization of membrane traffic and the regulation of Rabs themselves. In addition, it has the potential to identify organelle specific proteins, as the peripheral membrane proteins that are recruited by Rabs are likely to become dissociated during the fractionation procedures used to purify organelles biochemically. Affinity chromatography can identify many effectors simultaneously with the benefit that those in stable complexes should be isolated with their normal binding partners. This approach has been applied with great success to Rab5, but attempts to apply it in parallel to larger sets of human Rabs have had more limited success (Christoforidis et al., 1999, Kanno et al., 2010). Our studies in *Drosophila* on the binding of Rabs to Golgi coiled-coil proteins indicated that affinity chromatography could work well with *Drosophila* Rabs (Sinka et al., 2008). *Drosophila* have the advantage that they have a simpler set of Rabs than humans do, as they lack the expansion to families of paralogs that occurred during the evolution of vertebrates (Klöpper et al., 2012, Zhang et al., 2007). Nonetheless, they have close homologs of most human Rabs (and only a few Rabs that are insect specific), and many key events in membrane traffic appear well conserved between humans and flies. Therefore, we present here the outcome of parallel affinity purifications using a comprehensive set of Rabs from *Drosophila*. For a large subset of these Rabs, we found specific interactions with *Drosophila* orthologs of known mammalian effectors. In addition, we found dozens of proteins that showed similarly specific interactions, including known components of vesicle traffic, but also other proteins including those lacking a known function. We present here the entire data set, as well as validation of over 20 of the previously unreported interactions.

## Results

### Identification of Rab Interactors by Affinity Chromatography

**Figure 1:**
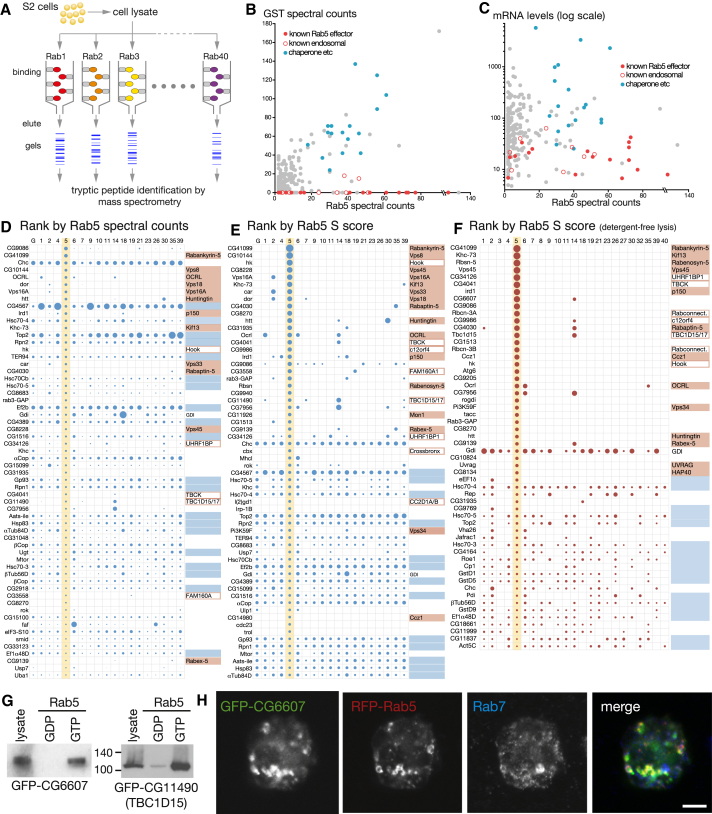
Analysis of the Rab5 Interactome to Test the Affinity Chromatography Approach (A) Schematic of the Rab effector isolation protocol. (B) Comparison of the spectral counts for proteins isolated by affinity chromatography with GST alone or GST-tagged GTP-locked Rab5. Colors indicate known Rab5 effectors, endosomal proteins not previously reported to bind Rab5, or typical nonspecific binders in affinity purifications (chaperones, cytoskeletal proteins, etc). (C) Plot of spectral counts versus mRNA levels for each of the Rab5 binding proteins. Coloring is as in (B). (D) Proteins ranked by the total number of spectral counts obtained with GST-Rab5 using detergent lysis, along with their spectral counts in other GST-Rab eluates. Circle area is proportional to the spectral counts. Only the top 70 proteins are shown, with the full list in Table S1A. Known Rab5 effectors (red), other proteins associated with endosomes (red frame), and typical nonspecific binders (blue) are highlighted. (E) Same as in (D), except that the data are not spectral counts but S scores as determined using the CompPASS method (Sowa et al., 2009). Only the top 70 proteins are shown (full list in Table S1B). The S score gives greater weight to proteins binding fewer Rabs and promotes known effectors. (F) Same as in (E), except S score for Rab effectors isolated using detergent-free lysis. (G) Affinity chromatography of lysates from S2 cells expressing GFP-CG6607 or GFP-CG11490 (dTBC1D15) using GDP- and GTP-locked Rab5. Blots were probed with antibodies against GFP. (H) Confocal micrographs of *Drosophila* S2 cells expressing both GFP-CG6607 and RFP-Rab5. Cells were stained with antibodies against Rab7. Scale bar, 5 μm. See also Figure S1 and Tables S1A and S1B.

*Drosophila melanogaster* has 27 Rabs, of which 23 have at least one mammalian ortholog, with these mammalian orthologs representing 50 of the 66 human Rabs (Figures S1A and S1B available online). All 23 *Drosophila* Rabs that have a mammalian ortholog were expressed as fusions to glutathione S-transferase (GST) with the Q→L mutation that is known in several Rabs to stabilize the GTP-bound form (Hyvola et al., 2006, Li and Stahl, 1993). They were coupled to glutathione Sepharose for affinity chromatography of lysates of *Drosophila* S2 cells, a widely used cell line thought to be from a macrophage-like lineage (Figure 1A). S2 cells were lysed in the detergent CHAPS, and the clarified lysate was applied to each GST-Rab column. After washing, proteins were eluted and separated on SDS gels. The gel lanes above the GST-Rab (∼45–50 kDa) were cut into sections and digested with trypsin, and peptides were sequenced by tandem mass spectrometry (Table S1).

To investigate the effectiveness of this approach, we examined the results for Rab5, the major Rab on early endosomes and the one that probably has the best characterized set of effectors from mammalian studies (Christoforidis et al., 1999). A variety of methods can be used to analyze mass spectrometry outputs from parallel isolations. Perhaps the simplest is to compare across all the baits the total number of tandem mass spectra that match peptides from a given protein (referred to here as “spectral counts”). Figure 1B plots the spectral counts for all proteins associated with GST-Rab5 against their counts from the material bound to GST alone (data appear in Table S1A). Most of the proteins that have high spectral counts with Rab5 and none with GST alone are *Drosophila* orthologs of known Rab5 effectors or are proteins that have not been reported to bind Rab5 but are known to be involved in endosomal function (discussed later). In contrast, typical nonspecific binders such as heat shock proteins and elongation factors showed similar spectral counts in both samples. Comparison of spectral counts to published mRNA expression data for the corresponding proteins revealed that known Rab5 effectors and endosomal proteins were expressed at much lower levels than the nonspecific binders, implying that they had been enriched to a much greater extent by Rab5, consistent with their being specific interactors (Figure 1C).

Since we examined many Rabs in parallel, our data also indicate the degree to which an interaction is specific to one particular Rab. Comparing the spectral counts for the proteins bound to Rab5 with those from other Rab columns showed that, for the known effectors and early endosomal proteins, few, if any, peptides were found in the eluates from other Rabs (Figure 1D). Various methods can be used to score hits from such multiple parallel interaction analyses. We used the S score from the CompPASS platform, which gives greater weight to interactions seen with fewer baits (Sowa et al., 2009). The S score worked well to promote known Rab5 effectors in the rankings, along with some of the known endosomal proteins (Figure 1E and Table S1B).

During optimization of cell lysis, we found that nonspecific binding of proteins from inside organelles could be reduced by lysing cells without detergent, with detergent only being added in the washes of the column. We thus generated a second data set using this approach, analyzing proteins both above and below the GST-Rab following gel separation, and also obtained a good yield of known Rab5 effectors (Figure 1F and Table S2). Although the data were cleaner, some known effectors were lost or reduced, which appeared, in at least some cases, to be due to poor solubilization in the absence of detergent (data not shown). Rab3, Rab8, Rab27, Rab32, and Rab40 were also included in this second data set but either expressed poorly or showed few specific effectors (Table S2), but for all other Rabs, we used the results from both data sets in our analysis (see Table S3 for the combined data).

### Interaction Partners Found with Rab5

Rab5 is on early endosomes and has been very well conserved in eukaryotic evolution. Studies on mammalian Rab5 have identified numerous effectors, and we found many of these in both data sets. These include *Drosophila* orthologs of Rabankyrin-5, Rabaptin-5, Rabex-5, Rabenosyn-5, huntingtin, Kif13A, Vps34, and Ccz1. In addition to known Rab5 effectors, we found several proteins whose mammalian orthologs are localized to endosomes but whose Rab5 association has not been reported. These include UHRF1BP1 (CG34126 in *Drosophila*), Hook, and its binding partners AKTIP (Cbx) and FAM160A2 (CG3558) (Otto et al., 2010, Xu et al., 2008). In addition, we found orthologs of mammalian proteins linked to endosomal function but whose localization has not been investigated. These include lethal giant discs (CC2D1A/B in humans), CG11490 (Rab7 GAP TBC1D15/17), and all three of the uncharacterized “novel” (as described by Collinet et al., 2010) proteins that were reported to affect endosome morphology when knocked down in a genome-wide RNAi screen (CG6607/Ccdc128, CG9986/C12orf4, and the TBC domain Rab GAP CG4041/TBCK; Collinet et al., 2010). Finally, there were some conserved proteins that have no known function, such as Rodgi and CG8270, the *Drosophila* ortholog of human C18orf8.

Most of the Rab columns, including Rab5, bound GDI, the protein that extracts GDP-bound Rabs from membranes. This likely reflects incomplete GTP loading of the columns and thus highlights the fact that Rab-specific interactors will include true effectors and, potentially, some proteins that prefer the GDP-bound state. To test whether the aforementioned proteins are likely to represent Rab5 effectors, i.e., preferentially bind Rab5-GTP, we selected CG6607 (Ccdc128) and CG11490 (TBC1D15/17). Both were expressed in S2 cells as GFP fusions, and affinity chromatography showed that they preferentially bound to GTP-bound Rab5, with CG6607 also colocalizing with Rab5 in S2 cells (Figures 1G and 1H). In addition, antibodies to endogenous Hook confirmed it to be specifically enriched on the Rab5 column (Figure S1C). Taken together, these results show that our approach has successfully identified many bona fide Rab5 effectors, including some not previously reported. This suggested that we would find effectors for at least some of the other Rabs, so we examined the sets of proteins found associated with each Rab.

### Rab2 Interactors Provide Evidence for Golgi and Endosomal Functions

Rab2 is widely conserved in evolution, being present in mammals, plants, and protozoa. It has been lost from a few organisms, including budding yeasts, and its role is not entirely clear. It is found on the Golgi apparatus and has been proposed to have a role in ER-to-Golgi traffic or in the formation of secretory granules (Edwards et al., 2009, Short et al., 2001). Deletion of Rab2 from *C. elegans* perturbs secretory granule formation and the maturation of phagosomes, suggesting a role in the endocytic pathway (Guo et al., 2010, Sumakovic et al., 2009). Some Rab2 effectors have been identified, all of which are known Golgi proteins. These include golgin-45, and ICA-69 a BAR domain protein (Short et al., 2001). Their function is unknown, but *C. elegans* ICA-69 is also required for the formation of secretory granules (Edwards et al., 2009). Rab2 has also been reported to bind to the Golgi coiled-coil protein complex GM130/p115 (Short et al., 2001), and our previous studies found that *Drosophila* Rab2 binds to further golgins, GMAP, dGCC88, dGCC185, and dGolgin-245 (Sinka et al., 2008).

**Figure 2:**
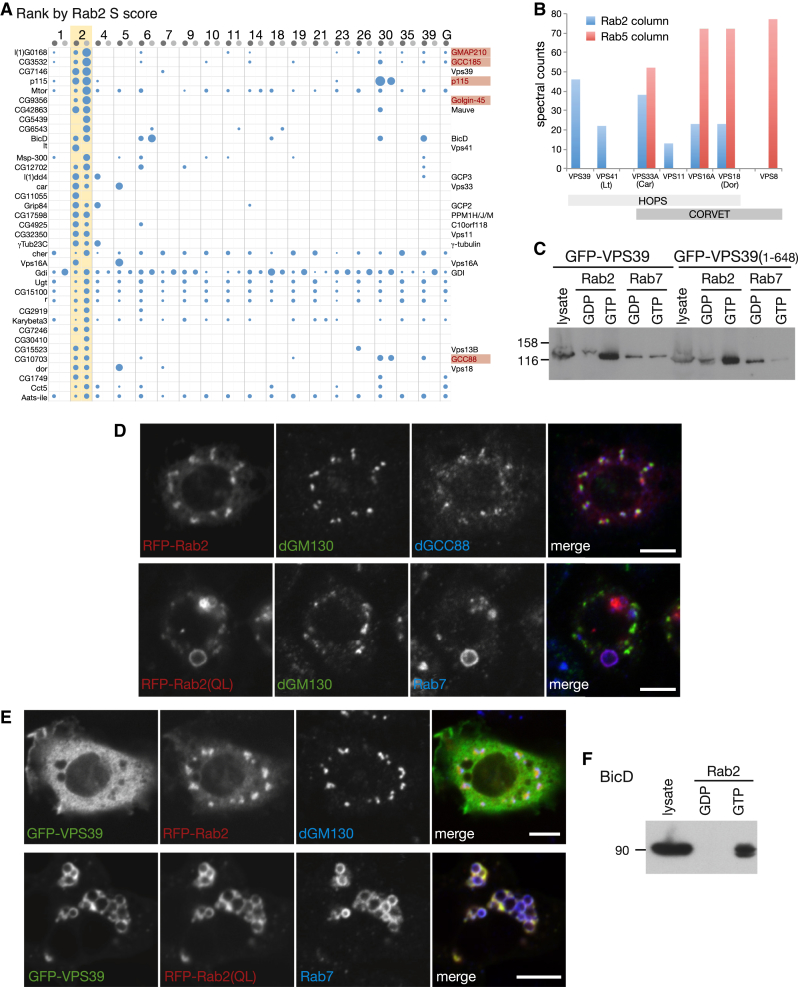
Rab2 Interactors Have Roles in the Golgi and in Endosomes (A) Proteins isolated from cell lysates as ranked by S-score for interaction with GST-Rab2 (detergent-free, lighter gray). Known Rab2 effectors are marked in red, and other proteins with links to membrane traffic are indicated. The top 36 are shown (full list, including CG9590 and CG32485, in Tables S1, S2, and S3). (B) Spectral counts of orthologs of the subunits of yeast CORVET and HOPS complexes from GST-Rab2 and GST-Rab5 columns using detergent lysates. The yeast CORVET subunit VPS6/VAM6 is not found outside of fungi. (C) Affinity chromatography of lysates from cells expressing GFP-VPS39 or GFP-VPS39 (amino acids 1–648) with GDP- and GTP-locked Rab2 and Rab7. Blots were probed with anti-GFP antibodies. (D) Confocal micrographs of *Drosophila* S2 cells expressing RFP-Rab2 or the GTP-locked mutant protein, RFP-Rab2(QL), and costained with dGM130 (*cis*-Golgi), dGCC88 (*trans*-Golgi), and Rab7 (late endosomes) antibodies as indicated. (E) Confocal images of *Drosophila* S2 cells coexpressing GFP-VPS39 and RFP-Rab2 or RFP-Rab2(QL). Cells were stained with antibodies against dGM130 or Rab7 as indicated. (F) Affinity chromatography of S2 cell lysate using GDP- and GTP-locked Rab2. Blots were immunoprobed for BicD. Scale bars, 5 μm. See also Figure S2 and Tables S1, S2, and S3.

Five of these seven known effectors showed highly specific interactions with Rab2 in both sets of conditions, and a sixth, dGolgin-245, was found in one set (Figure 2A). The exception is ICA-69 (CG10566), whose mRNA is expressed at the lowest levels in S2 cells, being primarily expressed in the nervous system. In addition, many other proteins bound specifically to the Rab2 column, including those with a role in membrane traffic and others previously uncharacterized.

#### VPS39 and the HOPS Complex

Both sets of conditions produced subunits of the HOPS complex that is found on endosomal membranes and acts in membrane tethering and fusion. In yeast, a second complex called CORVET shares four subunits with HOPS, with each complex having additional unique subunits. CORVET binds to yeast Rab5 via a CORVET-specific subunit, Vps8, but nothing has been reported about Rab interactions in metazoans or even whether the subunits form this pair of complexes (Balderhaar and Ungermann, 2013). Notably, four of the shared subunits were also found with Rab5 using detergent lysis conditions. However, the pattern of additional subunits was not the same for the two Rabs. Vps39 and Vps41 were only found with Rab2, while Vps8 was found only with Rab5 (Figure 2B). Strikingly, the Rab2 subunit set corresponds to those found in HOPS rather than CORVET, indicating that the two distinct tethering complexes found in yeast are conserved in metazoans and that HOPS interacts with Rab2 while CORVET interacts with Rab5. Of the two HOPS-specific subunits, the largest number of spectra was found with Vps39, and so it seemed a good candidate to interact with GTP-bound Rab2, and this was confirmed by yeast two-hybrid and in vitro binding (Figures 2C and S2A–S2C).

An interaction between Golgi-localized Rab2 and an endosomal tether may seem surprising, but it provides a possible explanation for the effects of Rab2 deletion on phagocytosis in *C. elegans* (Guo et al., 2010). Notably, although Rab2 was on the Golgi when expressed in S2 cells, when it was expressed as a GTP-locked form (Rab2Q65L), we observed large swollen structures which colocalized with the late endosomal marker Rab7 (Figure 2D). The Rab2Q65L could recruit Rab2 effectors to these structures, including Vps39, and we were able to use this to map the interaction to the N-terminal region predicted to form a β-propeller (Figures 2E, S2D, and S2E). We speculate that Rab2 can traffic on carriers from Golgi to endosomal structures, with GTP hydrolysis being required for its release from endosomes.

#### BicaudalD

The dynein adaptor BicaudalD (BicD) is a known effector for Rab6 (Short et al., 2002) but was also in both sets of Rab2 eluates. The interaction with Rab2 was GTP specific, suggesting that Rab2 also contributes to the recruitment of minus-end-directed motors to the Golgi (Figure 2F). BicD also showed GTP-dependent binding to Rab30 and to the Rab2 relative Rab39 (discussed later).

#### Uncharacterized Proteins CG4925, CG9590, and CG32485

**Figure 3:**
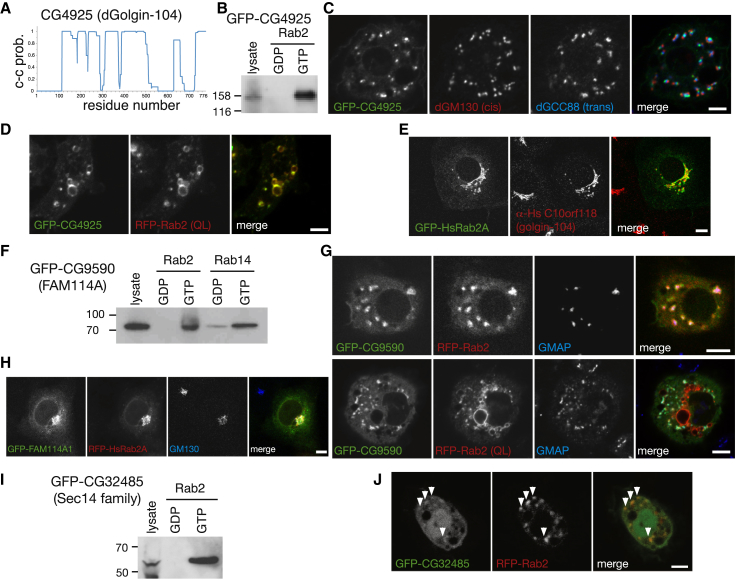
Interaction of Rab2 with Proteins of the Golgi Apparatus (A) Predicted coiled-coil (c-c prob.) in the CG4925 protein using COILS. (B) Affinity chromatography of S2 lysates from cells expressing GFP-CG4925 using locked versions of GST-Rab2. Blots were probed with anti-GFP antibodies. (C) Confocal micrographs of S2 cells expressing GFP-CG4925. Cells were stained with antibodies against the *cis*- (dGM130) and *trans*- (dGCC88) Golgi. (D) Confocal micrographs of live cells expressing GFP-CG4925 and RFP-Rab2(QL). (E) Micrographs of COS cells expressing GFP-Rab2A and immunolabeled with antibodies against C10orf118. (F) Affinity chromatography of S2 cell lysates expressing GFP-CG9590 with Rab2 GDP- and GTP-locked mutants. Blots were probed with antibodies against the GFP tag. (G) Fluorescent images of *Drosophila* S2 cells coexpressing GFP-CG9590 and either Rab2- or GTP-locked Rab2 fused to RFP. Cells were labeled with the *cis*-Golgi marker GMAP. (H) COS cells coexpressing RFP-Rab2A and one of the mammalian orthologs of CG9590, FAM114A1, tagged with GFP. Cells were stained for dGM130. (I) Immunoblot of lysate and eluates from a representative affinity chromatography experiment of S2 lysate expressing GFP-CG32485 (Sec14) using Rab2 nucleotide-locked mutants. The blot was probed with anti-GFP antibodies. (J) Live S2 cells expressing GFP-Sec14 and RFP-Rab2. Weak but reproducible colocalization was observed and is illustrated in the structures marked by the white arrowheads. Scale bars, 5 μm (10 μm in E and H). See also Figure S3.

Several uncharacterized proteins were found enriched in the Rab2 eluate and could be confirmed as effectors:

CG4925 is the *Drosophila* ortholog of human C10orf118 with both proteins predicted to be primarily coiled-coil (Figure 3A). It bound the GTP form of Rab2 by affinity chromatography and yeast two-hybrid, via the C-terminal 200 residues (Figures 3B, S3A, and S3B). Epitope-tagged CG4925 localized to the *trans*-Golgi in S2 cells and relocalized to the enlarged endosomes induced by overexpression of Rab2Q65L (Figures 3C and 3D). The mammalian protein is also on the Golgi and binds Rab2 and the Golgi via its C-terminal region (Figures 3E and S3C). Given that extensive coiled-coils and C-terminal attachment via a G protein are typical for the golgin coiled-coil proteins, we propose that this protein is named golgin-104/dGolgin-104 in humans and flies.

CG9590 is the *Drosophila* ortholog of FAM114A1/2, two closely related human proteins of unknown function. Screening a panel of Rabs by yeast-two hybrid showed binding to Rab2 and a weaker interaction with Rab14 (Figures S3D and S3E). GFP-tagged CG9590 localized to the Golgi in S2 cells and bound the GTP-form of Rab2 and, more weakly, Rab14 by chromatography (Figures 3F and 3G). It did not relocalize to the Rab2Q65L-induced swollen endosomes, suggesting that its membrane recruitment is stabilized by additional interactions (Figure 3G). GFP-tagged human FAM114A1 and FAM114A2 were also Golgi localized (Figure 3H; data not shown).

CG32485 is a member of the CRAL-TRIO (or Sec14) family that binds lipids and other hydrophobic molecules. It lacks a clear mammalian ortholog but has close relatives in plants and fungi. A Rab2-GTP-specific interaction was confirmed by affinity chromatography and yeast two-hybrid interactions (Figures 3I and S3F), and a GFP-tagged form of the protein showed faint but reproducible Golgi staining (Figure 3J).

Investigating every protein enriched on the Rab2 column was beyond our scope, but for those looking at the list of unconfirmed interactions, we would highlight CG15523 (*Drosophila* Vps13B), the TBC domain Rab GAP CG5337 (*Drosophila* TBC1D16), and the BEACH-domain family member Mauve (CG42863) as all binding Rab2 in both data sets and being known components of membrane traffic.

### Rab4 Interactors Reveal a Second Form of GARP Complex

**Figure 4:**
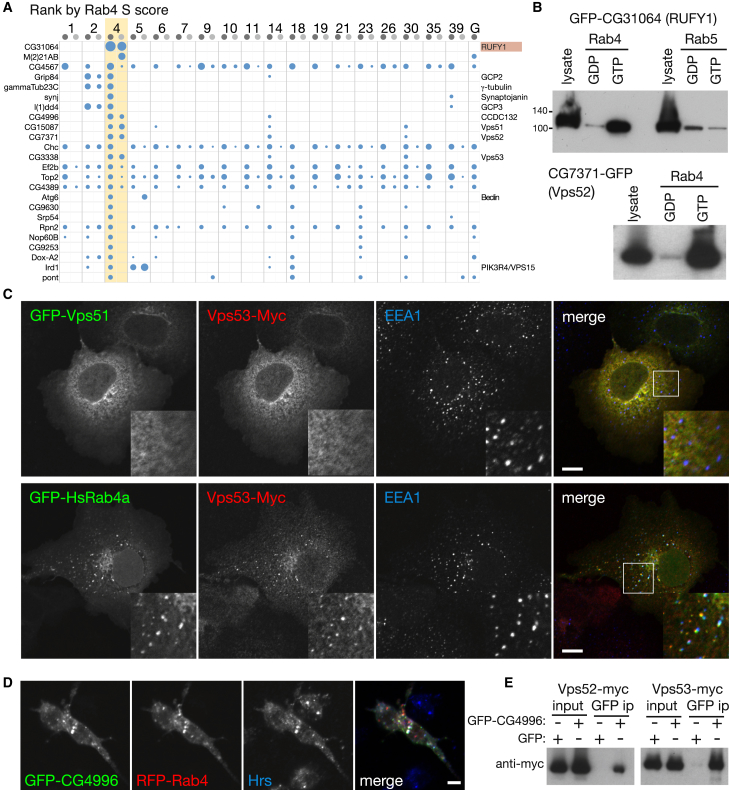
Rab4 Interacts with a Second Form of the GARP Complex (A) Proteins isolated from cell lysates ranked by S score for interaction with GST-Rab4 (detergent-free, lighter gray). The top 24 are shown (full list in Table S3). Known Rab4 effector marked in red, and other proteins with links to membrane traffic are indicated. (B) Anti-GFP immunoblot of affinity chromatography of lysates from S2 cells expressing GFP-CG31064 (FYVE domain form) or CG7371-GFP using GDP- and GTP-locked Rabs. (C) Confocal micrographs of COS cells coexpressing VPS51-GFP and VPS53-myc (upper panels) and GFP-Rab4A and VPS53-myc (lower panels). The proteins are human, and cells were stained for the early endosomal marker EEA1. (D) Fluorescent images of S2 cells coexpressing GFP-CG4996 and RFP-Rab4. Cells were immunolabeled with Hrs antibodies. (E) Immunoblots with anti-myc of GFP-Trap precipitations from extracts of S2 cells cotransfected with GFP-CG4996 or GFP, and with CG7371-Myc (Vps52) or CG3338-Myc (Vps53). GFP-Trap (ChromoTek) was used according to manufacturer’s instructions. Inputs are 10%. Both Vps51 and Vps53 associate with CG4996. ip, immunoprecipitation. Scale bars, 5 μm (D) or 10 μm (C). See also Figure S4 and Table S3.

Rab4 is widely conserved in evolution but has been lost in various lineages including budding yeasts. It is localized to endosomes and is proposed to play a role in recycling back to the surface. The best characterized Rab4 effector is RUFY1 (or Rabip4), one of three closely related paralogs (Cormont et al., 2001). CG31064, the *Drosophila* ortholog of these proteins, showed a strong and highly specific interaction with Rab4 under both lysis conditions (which we confirmed to be GTP-specific using a GFP-tagged CG31064), indicating that the Rab4-GTP was functional (Figures 4A and 4B). In addition to CG31064, there were several other proteins that were specific to Rab4 that have not previously been reported to be effectors. Most striking were Vps51, Vps52, and Vps53, three subunits of the GARP tethering complex that is found on the Golgi and involved in retrograde traffic from endosomes (Bonifacino and Hierro, 2011). These were found with or without detergent, and we were able to confirm that GARP subunit binding is GTP specific (Figure 4B). We also examined a GARP subunit (Vps53) in mammalian cells and found that it could be relocated to endosomes by overexpression of Rab4 (Figure 4C).

In addition to Vps51-53, GARP in mammals, worms, and yeast contains a fourth subunit, Vps54. This protein is present in *Drosophila* and is expressed in S2 cells, but it was only observed in the Rab4 eluate in one data set and at a low level. However, we noticed that, in both data sets, there was a high scoring protein recovered at a level similar to that of Vps51-53 called CG4996, with the four proteins also showing an interaction with Rab14. CG4996 has not been previously characterized, but we noticed that it is distantly related to Vps54 over much of its length (Figure S4). GFP-tagged CG4996 colocalized with Rab4 in S2 cells and interacted with GARP subunits by coprecipitation (Figures 4D and 4E). These results suggest that the GARP complex exists in a second version in which Vps54 is replaced by CG4996, with the result that it can now interact with Rab4. CG4996 is conserved from humans (Ccdc132) to plants and protozoa, indicating that this is an ancient divergence. We suggest that CG4996/Ccdc132 be named Vps54L and the second complex be named GARPII.

Other Rab4-specific proteins were not characterized, but the higher hits include synaptojanin, a lipid phosphatase involved in endocytosis, and CG43367, the *Drosophila* ortholog of the human BEACH domain proteins NBEAL1 and NBEAL2, which have a role in the formation of lysosome-related organelles.

### Rab6 Effectors

**Figure 5:**
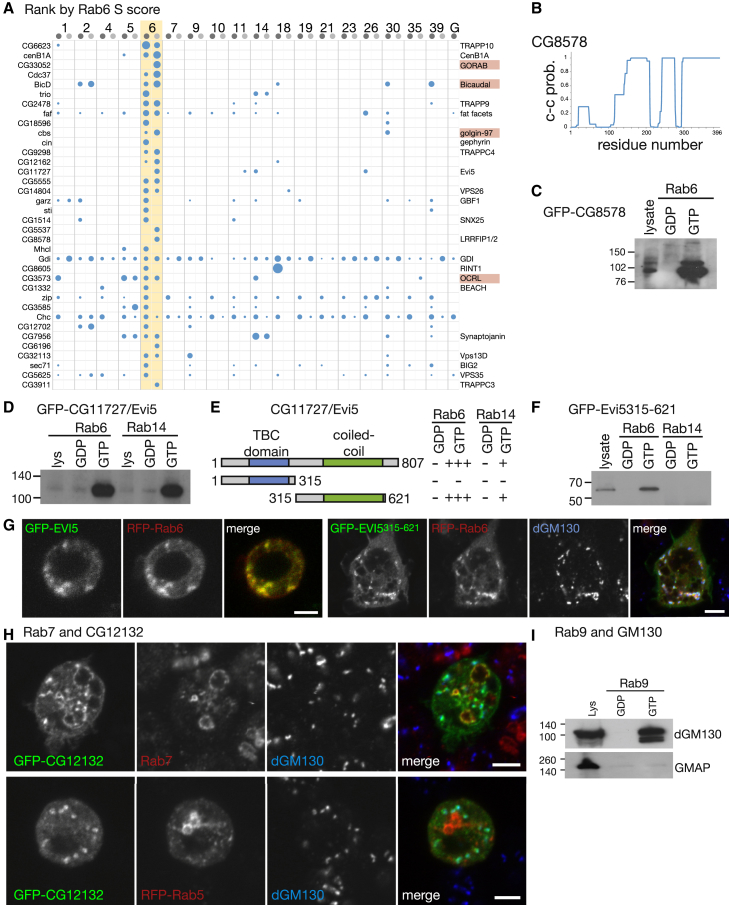
Binding Partners of Rab6, Rab7, and Rab9 (A) Proteins isolated from cell lysates ranked by S score for interaction with GST-Rab6 (detergent-free, lighter gray). The top 36 are shown (full list in Table S3). Known Rab6 effectors are marked in red, and other proteins with links to membrane traffic are indicated. (B) Predicted propensity for CG8578 to form coiled-coil along its length. (C) Anti-GFP immunoblot of affinity chromatography of S2 cell lysates expressing GFP-CG8578 using GDP- and GTP-locked versions of Rab6. (D) Interaction of Evi5 with Rab6 and Rab14 confirmed by affinity chromatography with lysates from cells expressing GFP-Evi5. (E) Summary of yeast two-hybrid assays with Evi5 truncations as prey and GDP- and GTP-locked version of Rab6 and Rab14 as bait. (F) Immunoblot of lysates and eluates from an affinity chromatography experiment of lysates prepared from GFP-Evi5 (315-621)-expressing cells using both Rab6- and Rab14-locked mutant proteins. The blot was probed with anti-GFP antibodies. (G) Live cells coexpressing GFP-Evi5 and RFP-Rab6 (left hand panels) and confocal micrographs of fixed cells coexpressing a C-terminal fragment of Evi5 (315-621aa) with RFP-Rab6 (right panels). The latter are immunolabeled with dGM130 antibodies. The N-terminal fragment (1–315) did not bind Rab6 in vitro and was diffuse in cells (data not shown). (H) Upper panels show confocal images of cells expressing GFP-CG12132, costained with anti-Rab7 antibodies (late endosomes) and anti-dGM130 antibodies (Golgi). Lower panels show GFP-CG12132 localization is distinct from RFP-Rab5 (early endosomes) in S2 cells. (I) Affinity chromatography of S2 cell lysates using nucleotide-locked versions of Rab9. Blots were probed with antibodies against golgins dGM130 and GMAP. Scale bars, 5 μm. See also Figure S5 and Table S3.

Rab6 is widely conserved among eukaryotes. Localized to the *trans*-Golgi, it is involved in retrograde traffic from endosomes and possibly exit from the *trans*-Golgi network and retrograde trafficking from Golgi to ER. Known Rab6 effectors include the dynein adaptor BicaudalD; the lipid phosphatase OCRL1; the golgins TMF1, golgin-97, GCC88, and GCC185; the putative Rab GEF DENND5; and GORAB/SCYL1BP1 (Hutagalung and Novick, 2011). *Drosophila* orthologs of five of these proteins were found associated with high specificity to the Rab6 column, being BicD, CG3573 (OCRL1), CG33052 (GORAB), dGolgin-97, and dGCC88 (Figure 5A). The Rab6 column also bound specifically other components of membrane traffic and some proteins whose function is less clear.

#### CG8578, a Coiled-Coil Protein of Unknown Function

CG8578 is the *Drosophila* ortholog of two human proteins (LRRFIP1/LRRFIP2), with all three proteins predicted to be coiled-coil over much of their length (Figure 5B). LRRFIP1/2 have been reported to have a regulatory role in toll-like receptor signaling, although their function is unknown (Dai et al., 2009). CG8578 showed Rab6 specific binding from the detergent-free lysate. A GFP-tagged form expressed in S2 cells showed robust GTP-specific binding to Rab6 and was localized to the Golgi apparatus (Figures 5C and S5A).

#### The Rab GAP Evi5

CG11727 is the *Drosophila* ortholog of the Rab GAP Evi5 (and its paralog Evi5L) that has been reported to act on Rab11 and, possibly, other Rabs (Westlake et al., 2007). A GFP-tagged form of CG11727 expressed in S2 cells bound to Rab6-GTP (Figure 5D). Since the protein contains a TBC Rab GAP domain, it was possible that this was responsible for the interaction with Rab6. However, Rab6-GTP binds to the C-terminal coiled-coil region of the protein (Figures 5E and 5F), and this C-terminal region was sufficient for Golgi localization (Figure 5G). We also observed an interaction with Rab14 in the detergent lysate, which we could confirm, but in vitro binding to the C terminus was less pronounced, and in S2 cells, GFP-Evi5 was not directed to Rab14-coated endosomes (Figures 5E and 5F; Figure S5B). This indicates that the Rab11 GAP, CG11727/Evi5, is an effector of Rab6 and, possibly, Rab14; hence, it appears to be another example of crosstalk between Rabs (Grosshans et al., 2006).

#### The TRAPP Complex

The TRAPP complex is found on the Golgi apparatus and is an exchange factor for Rab1 and, potentially, Rab11. It has a core of six to seven small subunits plus several additional large subunits, and in metazoans, it exists in at least two versions that have different sets of large subunits (Bassik et al., 2013). Eight TRAPP subunits showed highly specific Rab6 binding from detergent-free lysate, being six of the seven core subunits and both of the larger subunits that define TRAPPII (TRAPPC9 and TRAPPC10) but none of the four TRAPPIII-specific subunits (Bassik et al., 2013). In addition, the detergent data set contained all three of the TRAPPII subunits larger than the ∼45 kDa size minimum for these data. Thus, it appears that Rab6 interacts specifically with the metazoan TRAPPII complex.

Of the other Rab6-specific interactors, Fat facets was found in both data sets and is a deubiquitinase that acts on a Golgi-localized coat adaptor (LqfR, *Drosophila* CLINT1) and whose mammalian ortholog FAM/USP9X has been reported to be Golgi localized (Lee et al., 2009, Murray et al., 2004). The *Drosophila* orthologs of the membrane traffic component Vps13D (CG32113), the Inpp5f phosphoinositide phosphatase (CG7956), and some subunits of the retromer complex were also found in both data sets.

### Rab7 and Rab9 Effectors

Rab7 is conserved in most eukaryotes and is localized to late endosomes. A duplication event during the emergence of metazoans created Rab9, which is also on endosomes. Previously reported effectors of Rab7 include the dynein adaptor RILP and the two related proteins PLEKHM1 and Rubicon (Tabata et al., 2010). Of these, *Drosophila* PLEKHM1 (CG6613) was found in both data sets, and Rubicon (CG12772) was found in one, suggesting that the GST-Rab7 was functional, and we confirmed that the interaction with CG6613 was GTP specific (Figures S5C and S5D). There were not many other high-scoring hits shared by both data sets, although one was CG12132 (c11.1), a HEAT repeat protein with several mammalian paralogs of unknown function. A GFP-tagged form of CG12132 localized to both the Golgi and Rab7-positive late endosomes, but not early endosomes (Figure 5H), and we could detect a GTP-specific interaction by yeast two-hybrid, although not by affinity chromatography (data not shown). The putative Rab7 interactors that we did not test include *Drosophila* orthologs of Spg11 and Spg15, human spastic paraplegia proteins that form a complex on late endosomes (Hirst et al., 2013).

A range of proteins showed specific binding to the Rab9 column (Figure S5E). These did not include the two reported Rab9 effectors (SGSM1/2) that have clear *Drosophila* orthologs (CG1695/CG32506), although these are primarily expressed in the nervous system and not in S2 cells. However, we did observe that the Golgi coiled-coil protein dGM130 bound Rab9 in addition to Rab2 and Rab30, whose binding had been previously reported (Sinka et al., 2008). This interaction could be confirmed as GTP specific using both affinity chromatography and yeast two-hybrid and was mapped to a central region of the protein (Figures 5I and S5F).

Of the interactors not investigated, perhaps of most note is CG14299, which bound to both Rab7 and Rab9. This is the *Drosophila* ortholog of Epg-5, a protein involved in autophagy and endocytosis in *C. elegans* and mammals (Zhao et al., 2013). In addition, Rab9 showed specific binding to the *Drosophila* orthologs of Munc13-4 (CG11819), the lipid phosphatases INPP4A/B (CG42271), and the Rab5 GAP SGSM3 (CG12241) and shared with Rab6 an association with two members of the Vps13 family, which has an unknown role in endosomal function.

### Rab18 Binds to an ER Tethering Complex and to Proteins Linked to Disease in Humans

Rab18 is conserved from humans to plants and is absent from only a few species, including a subset of budding yeasts. Its function is enigmatic, with evidence for both a location to lipid droplets and a role in traffic in the ER-Golgi circuit, but no effectors have been reported (Dejgaard et al., 2008, Ozeki et al., 2005). Mutation of the single *Rab18* gene in humans causes the recessive developmental disorder Warburg Micro syndrome (Bem et al., 2011). Two other Warburg genes, *RAB3GAP1* and *RAB3GAP2*, encode the two subunits of Rab3GAP, which was reported to act on Rab3 but has recently been shown to be a GEF for Rab18 (Gerondopoulos et al., 2014).

**Figure 6:**
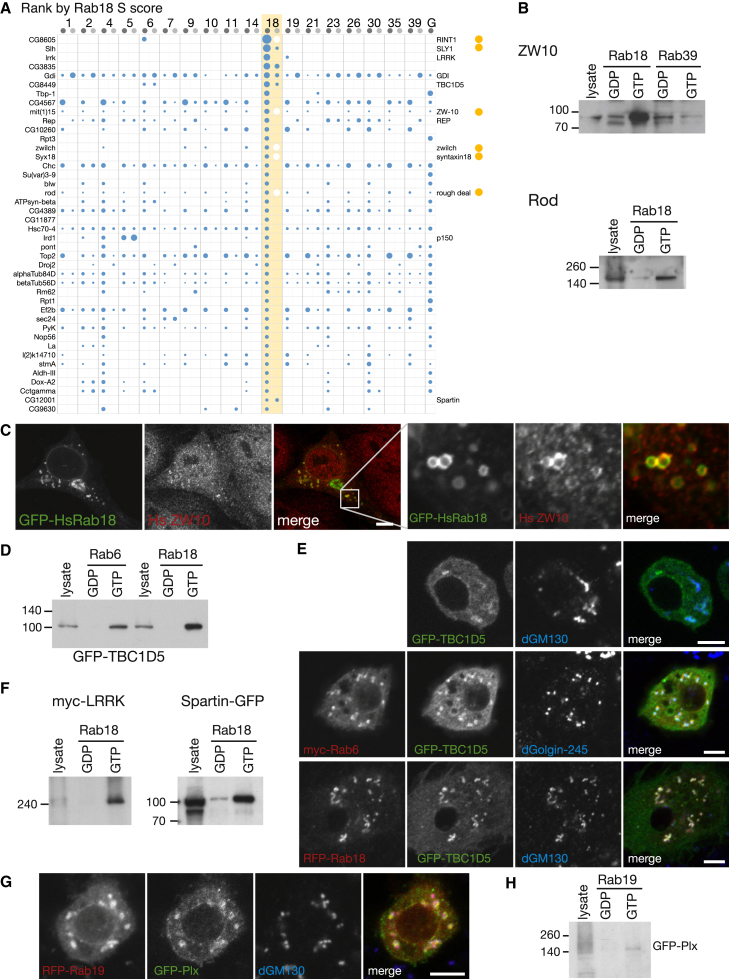
Rab18 Has Roles at the ER and the Golgi (A) Proteins isolated from cell lysates ranked by S score for interaction with GST-Rab18 (detergent-free, lighter gray). Subunits of the NRZ/Dsl1 complex (yellow dots), and other proteins with links to membrane traffic are indicated. The NRZ/Dsl1 complex contains two membrane-spanning proteins and so would not be isolated by the detergent-free approach (white circles). The top 42 S scores are shown with the full list, including the *Drosophila* orthologs of Rab3GAP, in Table S3. (B) Affinity chromatography of S2 cell lysate with Rab18. Blots were probed with either anti-ZW10 antibodies or anti-Rod antibodies as indicated. (C) Confocal micrographs of COS cells expressing GFP-HsRab18 and stained with antibodies against endogenous ZW10. The ZW10 accumulates around the Rab18-positive lipid droplets. (D) Affinity chromatography by Rab6 and Rab18 of lysates prepared from S2 cells expressing GFP-TBC1D5. (E) Fluorescent images of GFP-TBC1D5 expressed in S2 cells alone (top panels) or with myc-Rab6 (middle panels) or RFP-Rab18 (lower panels). Cells were stained with the Golgi antibodies dGM130 or dGolgin-245 as indicated. Expression of Rab18 enhances recruitment of TBC1D5 to the Golgi. (F) S2 cells expressing LRRK2-myc or Spartin-GFP were subjected to affinity chromatography with locked forms of Rab18. The resulting blots were probed for the relevant tag. (G) Fluorescent images of RFP-Rab19 and GFP-Plx expressed in S2 cells. Cells were stained for the Golgi marker dGM130. (H) Affinity chromatography by Rab19 of lysates prepared from S2 cells expressing GFP-Plx. Scale bars, 5 μm (10 μm in C). See also Figure S6 and Table S3.

Several proteins bound with high specificity to Rab18 (Figure 6A). These include subunits of the Dsl1 complex (or NRZ in mammals), which is localized to ER membranes and tethers and then fuses vesicles returning from the Golgi (Civril et al., 2010, Wainman et al., 2012). These subunits (Zw10, Rod, Zwilch, RINT1, and syntaxin-18) were only recovered from the detergent lysate, consistent with syntaxin-18 being a SNARE with a transmembrane domain. The only subunits not detected were two further SNAREs, which are below the ∼45 kDa minimum of this data set. Antisera against two NRZ subunits confirmed the interaction with Rab18 and showed it to be GTP specific (Figure 6B). In S2 cells, red fluorescent protein (RFP)-Rab18 colocalized with ER and early Golgi, and with GFP-tagged ZW10 (Figures S6A and S6B). These results suggest that Rab18 may assist the tethering of COPI-coated vesicles to the ER. In mammalian cells, Rab18 localizes to the ER and Golgi, but when overexpressed, it accumulates on lipid droplets (Ozeki et al., 2005). When we overexpressed Rab18 in mammalian cells, the endogenous NRZ subunits shifted from a diffuse distribution to being clustered around lipid droplets (Figures 6C and S6C). In addition, when human Rab18 was used for affinity chromatography of human cell lysates, subunits of NRZ were among the most abundant proteins showing GTP-specific binding (Figure S6D). The physiological relevance of overexpressed Rab18 being on lipid droplets is unclear, but these data at least indicate that its interaction with NRZ is relevant to mammalian cells and would explain why overexpression of Rab18 induces an association between lipid droplets and ER (Ozeki et al., 2005).

We also observed in both data sets an interaction between the putative Rab GAP CG8449 (TBC1D5) and both Rab18 and Rab6. The substrate of this GAP is unknown, but both Rabs showed a GTP-specific interaction by in vitro binding, and when overexpressed, they increased the recruitment of GFP-TBC1D5 to the Golgi (Figures 6D and 6E). The detergent data set also contained LRRK, the *Drosophila* ortholog of human LRRK2, the most common genetic determinant of Parkinson’s disease (Dodson et al., 2012). The role of this protein is unclear, but we could confirm a GTP-specific interaction with Rab18 (Figure 6F). Finally, Spartin, the *Drosophila* ortholog of the protein encoded by the spastic paraplegia gene *SPG20* was Rab18 specific in both data sets, and we confirmed that this interaction was GTP dependent (Figure 6F). Notably, one of the several roles proposed for Spartin is a function on lipid droplets (Renvoisé et al., 2012).

Several of the other Rab18 hits seem noteworthy. These include CG31935, the *Drosophila* ortholog of the Rab3GAP catalytic subunit whose human mutation causes the same Warburg syndrome as Rab18 mutations. This showed binding to Rab5 and Rab18 under both sets of conditions, and its regulatory subunit (CG7061/Rab3-GAP) was found with the same Rabs in one data set; human orthologs of both proteins bound specifically to human Rab18-GTP (Figure S6D).

### Rab19 Interacts with a Member of the TBC Family of Rab GAPs

Rab19 emerged in metazoans as an expansion from Rab1, and humans have two paralogs, Rab19 and Rab43, with the latter implicated in traffic between endosomes and Golgi but lacking known effectors. We found that Pollux (Plx) gave high specificity Rab19 binding in both data sets (Figure S6E). Plx is the *Drosophila* ortholog of the related human Rab GAPs TBC1D1 and TBC1D4/AS160 that regulate the insulin-controlled traffic of the glucose transporter GLUT4 and have been linked to obesity (Bogan, 2012). GFP-tagged Plx localizes to the Golgi along with Rab19 in S2 cells, and the interaction with Rab19 was GTP specific by affinity chromatography (Figures 6G and 6H).

### Rab30 Interacts with a Diverse Set of Golgi Proteins

**Figure 7:**
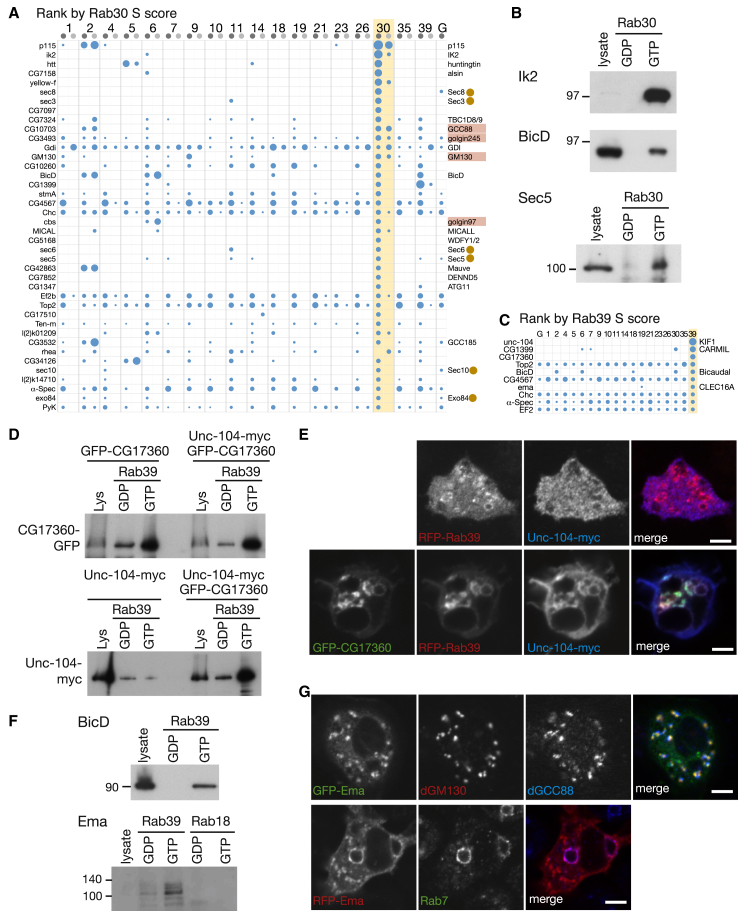
Rab30 Interacts with Golgi Proteins and Rab39 Recruits the Kinesin-3 Motor Unc-104 via an Adaptor (A) Proteins isolated from cell lysates ranked by S score for interaction with GST-Rab30 (detergent-free, lighter gray). The top 40 are shown (full list in Table S3). Known Rab30 effectors are marked in red, and other proteins with links to membrane traffic are indicated, including exocyst subunits (ochre dots). (B) S2 cell lysates were subjected to affinity chromatography with GDP- and GTP-locked Rab30. Blots were probed for IK2, BicD, or the exocyst subunit Sec5. (C) Proteins isolated from cell lysates prepared with detergent lysis ranked by S score for interaction with GST-Rab39. The top 10 are shown (full list in Table S1). Proteins with links to membrane traffic are indicated. (D) Affinity chromatography with GST-Rab39 of S2 cell lysates expressing GFP-CG17360 or Unc-104-myc alone, or the two in combination. Upper panel is probed to detect GFP-CG17360, while the lower panel is probed for the myc tag fused to Unc-104. (E) Confocal micrographs of cells expressing Unc-104-myc and RFP-tagged Rab39, with or without GFP-tagged CG17360. Only with the latter is Unc-104-myc enriched on Rab39-positive membranes. (F) Immunoblots of affinity chromatography of S2 cell lysate with nucleotide-locked Rab18 or Rab39, probed for endogenous BicD or Ema. (G) Confocal micrographs of cells expressing GFP- or RFP-tagged Ema and probed with antibodies against dGM130, dGCC88, and Rab7. Scale bars, 5 μm. See also Figure S7, Table S1, and Table S3.

Rab30 is widely conserved in metazoans, having expanded from Rab1. It is on the Golgi, but its precise role is unclear (Kelly et al., 2012). We previously found that *Drosophila* Rab30 binds to the golgins dGCC88, dGolgin-97, dGolgin-245, and dGM130 (Sinka et al., 2008). Of these four, three were high-specificity hits under both sets of conditions (along with p115, the binding partner of GM130), indicating that GST-Rab30 was functional (Figure 7A).

In addition to these known hits, we found in both data sets Ik2, one of two *Drosophila* orthologs of the mammalian IκB kinases that act in NF-κB signaling (Dubin-Bar et al., 2008). Affinity chromatography confirmed the interaction to be GTP specific (Figure 7B). Ik2 forms a complex with Spn-F, and both proteins have been reported to have a “punctate” distribution in S2 cells (Dubin-Bar et al., 2008), which we confirmed corresponds to the Golgi apparatus (Figure S7). Rab30 interactors from the detergent lysate included the dynein adaptor BicD (that also binds Rab6 and Rab2) and all eight subunits of the exocyst complex that mediates tethering of Golgi-derived vesicles at the plasma membrane, and we confirmed that both interactions were GTP specific (Figure 7B).

Further Rab30-specific hits that remain to be investigated include CG5168, the *Drosophila* ortholog of WDFY1/2 that play a role in endocytosis; CG7324, the ortholog of the mammalian TBC1D8/9 Rab GAPs; CG7852, the DENND5 Rab GEF; and all four subunits of the GARP complex (but not CG4996, the VPS54-like protein described earlier for Rab4).

### Rab39 Is Linked to Export from the Golgi

Rab39 is distantly related to Rab2 and appeared early in metazoan evolution. It has two paralogs in humans, with Rab39A being linked to caspase function, but its role in membrane traffic is unclear (Becker et al., 2009). The top hit with *Drosophila* Rab39 was Unc-104, the *Drosophila* ortholog of the kinesin-3 KIF1A (Figure 7C). We noticed that the third hit was CG17360, a member of the PLEKHM family, of which a mammalian member recruits kinesin-1 to lysosomes (Rosa-Ferreira and Munro, 2011). In vitro binding assays revealed GTP-dependent binding of GFP-CG17360 to Rab39 regardless of whether Unc-104 was coexpressed, whereas overexpressed Unc-104 only bound efficiently when GFP-CG17360 was also expressed (Figure 7D). In S2 cells, exogenous CG17360 localized with Rab39 on the Golgi and induced the Golgi accumulation of Unc-104 (Figure 7E). These results indicate that CG17360 acts as a linker to recruit Unc-104 to Golgi membranes or Golgi-derived carriers. Cargos for long-range transport can move bidirectionally, and the dynein adaptor BicD was also present in the Rab39 eluate and showed a GTP-specific interaction like that for Rab2 and Rab6 (Figures 2F and 7F).

We also found a high score for the protein Ema, mutations in which cause defects in endosomal function, with variants in the mammalian ortholog CLEC16A being linked to several autoimmune diseases (Hakonarson et al., 2007, Kim et al., 2012). Ema associated with GST-Rab39 in a GTP-dependent manner (Figure 7F), and GFP-tagged Ema was found on both the Golgi and late endosomes in S2 cells.

## Discussion

Identification of the effectors of Rabs is key to understanding cellular organization, and our results show that parallel affinity chromatography can do this effectively. We have found and validated interaction partners for 11 Rabs, and for Rab11, we found specific interactions with known effectors (dRip11 and Nuf). A further two Rabs showed strong and specific interactions with membrane traffic machinery that we did not validate because of a lack of suitable reagents: Rab26 with the spastic paraplegia proteins Spg11 and Spg15 (also found with Rab7), and Rab35 with the Rab GEF Sbf (SBF1/2 in humans). For the remaining nine Rabs, the lack of obvious specific interactions may have had several causes. In many cases, the recombinant Rabs may have been inactive, as they expressed with poor yield (Rab8, Rab10, Rab21, Rab23, Rab27, Rab32, and Rab40). In other cases, such as Rab3, the Rab is expressed predominantly in brain and at only very low levels in S2 cells (Jin et al., 2012; Figure S1B), so it is possible that their effectors are absent from S2 cells.

**Figure 8:**
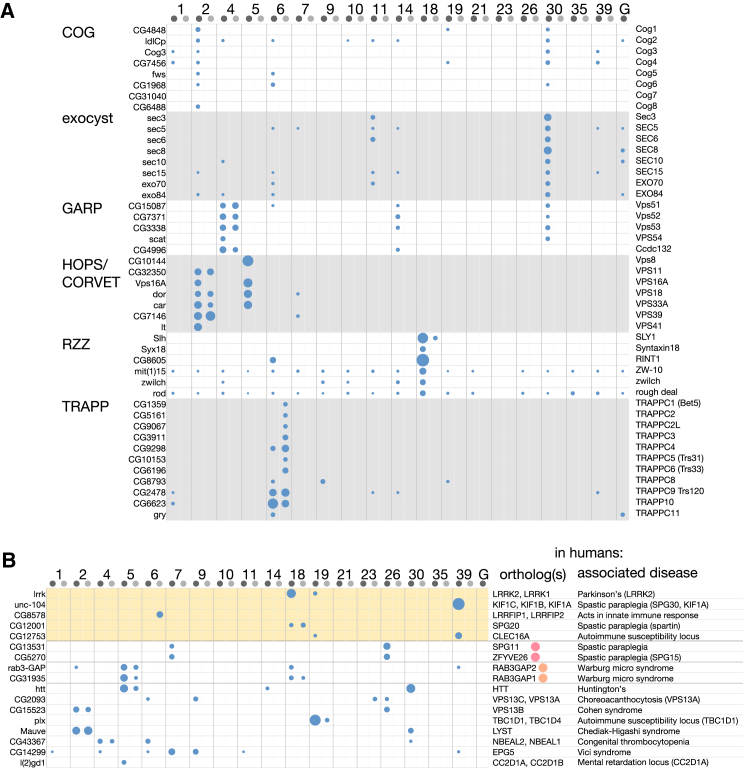
Rab Interactions with Tethering Complexes and with Proteins Associated with Disease (A) S scores for binding of the subunits of tethering complexes to GST (G) or the indicated GST-Rab fusions (detergent-free, lighter gray). Circle area is proportional to the S score. (B) Same as in (A) but for proteins whose human orthologs are linked to disease. Validated effectors are highlighted in yellow, and colored dots show subunits of known complexes. The latter show similar patterns of binding, increasing the likelihood that the identifications are correct.

One advantage of seeking effectors using affinity chromatography rather than yeast two-hybrid screens is that it allows isolation of intact protein complexes, and, in some cases, it may be that no one individual subunit can bind the Rab in isolation. This provides the opportunity to detect many, if not all, of the subunits of a complex rather than just that which binds the Rab, as illustrated for multisubunit tethering complexes in Figure 8A. The pattern of subunits isolated can reveal variants of particular complexes, such as our demonstration of GARPII and providing evidence for CORVET and HOPS coexisting in metazoans as well as yeast. However, a further consequence is that a protein that binds to a particular Rab column may not bind directly but instead via one of the other specific interactors, such as our finding of CG17360 acting as a linker between Rab39 and the kinesin Unc-104.

For a Rab whose function is unknown, the set of effectors should give a strong indication of the Rab's in vivo role. For instance, for Rab18, there are seemingly discrepant data for a role in the early Golgi or on lipid droplets (Dejgaard et al., 2008, Ozeki et al., 2005). The binding of Rab18 to the Dsl1/NRZ tethering complex would be consistent with retrograde traffic from early Golgi to ER, but Rab18 also interacted with Spartin, a protein linked to lipid droplet function, suggesting that Rab18 may have a role in tethering multiple structures to the ER. Another example is Rab2, which bound a range of Golgi proteins, consistent with previous studies, but also bound the HOPS complex that mediates tethering and fusion to endosomes (Peplowska et al., 2007). This suggests that, at least in some cell types, Rab2 is on carriers coming from the Golgi that fuse with endosomal compartments, hence providing an explanation for Rab2 acting on phagosomes in addition to the Golgi (Guo et al., 2010). In yeast, the HOPS complex binds to Ypt7 (Balderhaar and Ungermann, 2013), but we found only weak binding of two subunits to its ortholog Rab7 (Figure 8A). However, Rab2 is conserved in many eukaryotes but lost in budding yeasts, with the same being true for Arl8, another GTPase reported to bind to HOPS in metazoans (Garg et al., 2011). Therefore, it may be that, in the absence of these two GTPases, HOPS in yeast has become more reliant on binding Rab7/Ypt7.

The types of protein that we found as validated interactors are consistent with the general trends seen among the Rab effectors found in previous studies of individual Rabs (Hutagalung and Novick, 2011, Pfeffer, 2013, Stenmark, 2009). The large number of vesicle tethers is consistent with the notion that Rabs direct the initial contacts between transport carriers and their target organelle and, hence, are key determinants of the specificity of membrane traffic (Yu and Hughson, 2010). We also found interactions between Rabs and various Rab GAPs and GEFs. In some cases, these may be due to the Rab being recognized as a substrate, but at least in one case, we were able to show that the interaction is GTP dependent and outside of the regulatory domain (Rab6 binding to the ortholog of the Rab11 GAP Evi5). In addition, specific interactions were found with regulators of phosophoinositides, molecules that share with Rabs the function of recruiting specific proteins to organelles (Di Paolo and De Camilli, 2006).

Tethers, motors, and identity regulators are not the only classes of effector found in previous studies; consistent with this, we also found specific interactions with protein kinases and phosphatases. Further studies will be required to determine if such signaling molecules regulate membrane traffic or other organelle-specific processes. Notably, several of the previously unreported interactions that we found involved *Drosophila* orthologs of human proteins linked to disease (Figure 8B). Some of these disease proteins have unclear functions, such as LRRK, huntingtin, Spartin, and Clec16A, and investigation of their Rab interactions should, at the very least, reveal their cellular sites of action and so provide clues as to their in vivo roles.

It is likely that parallel affinity chromatography could be further improved by advances in protein expression, affinity chromatography, and mass spectrometric analysis. Even our current approach has provided a high yield of informative interactions; thus, it seems likely that future application to other cell types and species will reveal much about the organization of membrane traffic and how the basic themes are varied to generate the diverse cell types of multicellular organisms.

## Experimental Procedures

### GST-Rab Affinity Chromatography

GTP-locked forms of *Drosophila* Rab proteins were expressed in *E. coli* as fusions to GST and coupled to glutathione sepharose beads. The Rabs contained the Q → L mutations that stabilize members of this family in the GTP-bound state (Hyvola et al., 2006, Li and Stahl, 1993). Cell lysates were prepared from *Drosophila* S2 cells using the detergent CHAPS or by dounce homogenization without detergent. After clarification, the lysates were applied to the GST-Rab columns, and after washing and elution, the bound proteins were separated by SDS-PAGE. See Supplemental Experimental Procedures for details.

### Mass-Spectrometric Identification of Rab Interactors

Tryptic peptides obtained by in-gel digestion were identified using sequencing by tandem mass spectrometry. The proteins bound to different Rabs were compared using spectral counts to give an approximate measure of abundance. To score the significance of interactors, we used the S score from the CompPASS platform (Sowa et al., 2009). The S score assigns more confidence to proteins that interact with fewer baits (in this case, fewer Rabs) and is, thus, a measure of specificity. For each Rab, the bound proteins were ranked by the highest S score they showed with either CHAPS-based lysis or detergent-free lysis, and the data were displayed using the bubble blot function in Microsoft Excel. See Supplemental Experimental Procedures for details.

### Validation of Rab Effectors

Selected components of membrane traffic or proteins of unknown function were chosen for validation based on the availability of antibodies to the endogenous proteins or of full-length cDNAs that could be tagged and expressed from plasmids transfected into cells. These reagents were used for localization studies in cells or to test binding to GDP- and GTP-locked forms of Rabs using small-scale affinity chromatography. Some cDNAs were used for yeast two-hybrid analysis against a panel of *Drosophila* Rabs (Sinka et al., 2008). See Supplemental Experimental Procedures for details.

## Supplemental Information

Document S1. Supplemental Experimental Procedures and Figures S1–S7

Table S1. Mass Spectrometric Analysis of Proteins Bound to Rabs in the Detergent Data Set, Related to Figure 1 Proteins identified as associating with at least one Rab in the data set from lysates prepared with CHAPS. The spectral counts are in Sheet S1A, and the S scores are in Sheet S1B (see tabs at the bottom of the sheet). For each protein, the gene number, FlyBase gene number (FBgn), gene symbol, molecular weight, and relative mRNA levels in S2 cells are stated.

Table S2. Mass Spectrometric Analysis of Proteins Bound to Rabs in the Detergent-free Data Set, Related to Figure 1 Proteins identified as associating with at least one Rab in the data set from lysates prepared without detergent. The spectral counts are in Sheet S2A, and the S scores are in Sheet S2B (see tabs at the bottom of the sheet). For each protein, the gene number, FlyBase gene number (FBgn), gene symbol, molecular weight, and relative mRNA levels in S2 cells are stated.

Table S3. Merged Mass Spectrometric Analysis from Both Data Sets, Related to Figures 2, 4, 5, 6, 7, and 8 Proteins identified as associating with at least one Rab in either data set. The spectral counts are in Sheet S3A, and the S scores are in Sheet S3B (see tabs at the bottom of the sheet). For each protein, the gene number, gene symbol, human orthologs, and molecular weight are stated.

Document S2. Article plus Supplemental Information
