# Supplementary material for: Toward a Comprehensive Map of the Effectors of Rab GTPases
Source: Dev Cell. 2014 Nov 10;31(3):358–73. doi: 10.1016/j.devcel.2014.10.007 (PMC4232348; doi:10.1016/j.devcel.2014.10.007)
Supplement: Document S1. Supplemental Experimental Procedures and Figures S1–S7 [file mmc1.pdf]

Developmental Cell, Volume *31*

Supplemental Information

Toward a Comprehensive Map
# of the Effectors of Rab GTPases

Alison K. Gillingham, Rita Sinka, Isabel L. Torres, Kathryn S. Lilley, and Sean Munro

Figure S1

A

	LECA	Basal Metazoa	Droso	Humans
		Rab1	Rab1	Rab1a
				Rab1b
		Rab35	Rab35	Rab35
		Rab33		Rab33a
	Rab1			Rab33b
		Rab19	Rab19	Rab19
				Rab43
		Rab30	Rab30	Rab30
		RabX6	RabX6	
		Rab8	Rab8	Rab8a
				Rab8b
				Rab13
				Rab12
		Rab15		Rab15
		Rab10	Rab10	Rab10
		Rab3	Rab3	Rab3a
				Rab3b
Group I				Rab3c
				Rab3d
	Rab8	Rab26	Rab26	Rab26
				Rab37
		Rab27	Rab27	Rab27a
				Rab27b
		Rab34	RabX5	Rab34
				Rab36
		RabX4	RabX4	
		Rab45		Rab45
		Rab44		Rab44a
				Rab44b
		Rab18	Rab18	Rab18
		Rab40	Rab40	Rab40a
	Rab18			Rab40aL
				Rab40c
				Rab40d
		Rab5	Rab5	Rab5a
				Rab5b
	Rab5			Rab5c
				Rab17
	Rab21	Rab21	Rab21	Rab21
Group II		Rab22		Rab22a
	Rab22			Rab22b
		Rab24		Rab24
	Rab24	Rab20		Rab20
	RabX1	RabX1	RabX1	
		Rab7	Rab7	Rab7a
	Rab7			Rab7b
		Rab9	Rab9	Rab9a
				Rab9b
Group III	Rab23	Rab23	Rab23	Rab23
	Rab29	Rab29		
		Rab32	Rab32 (Ltd)	Rab32
	Rab32			Rab38
	Rab7L1	Rab7L1		Rab7L1
		Rab2	Rab2	Rab2a
				Rab2b
	Rab2	Rab39	Rab39	Rab39a
				Rab39b
				Rab42
Group IV		Rab4	Rab4	Rab4a
	Rab4			Rab4b
		Rab11	Rab11	Rab11a
	Rab11			Rab11b
				Rab25
	Rab14	Rab14	Rab14	Rab14
		Rab6	Rab6	Rab6a
				Rab6b
Group V	Rab6			Rab6c
				Rab41
	Rab28	Rab28		Rab28
Group VI	RabL4	RabL4		RabL4

B

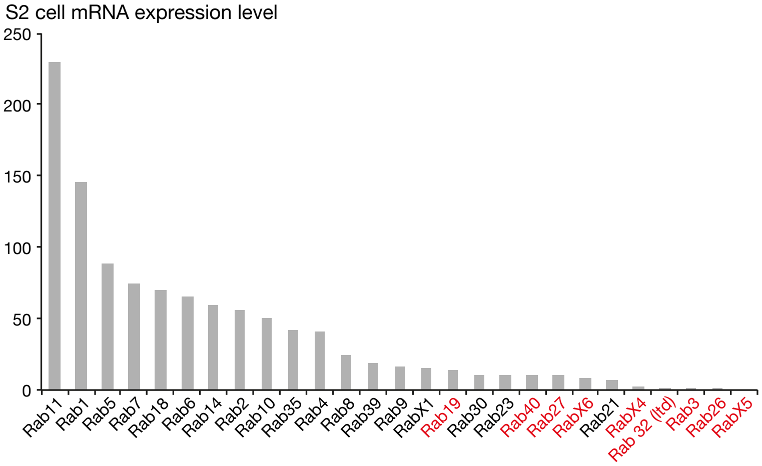

C

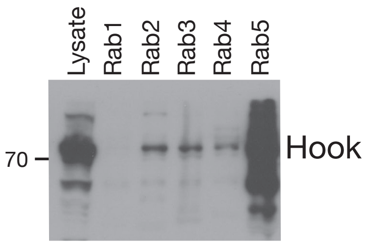

Figure S2

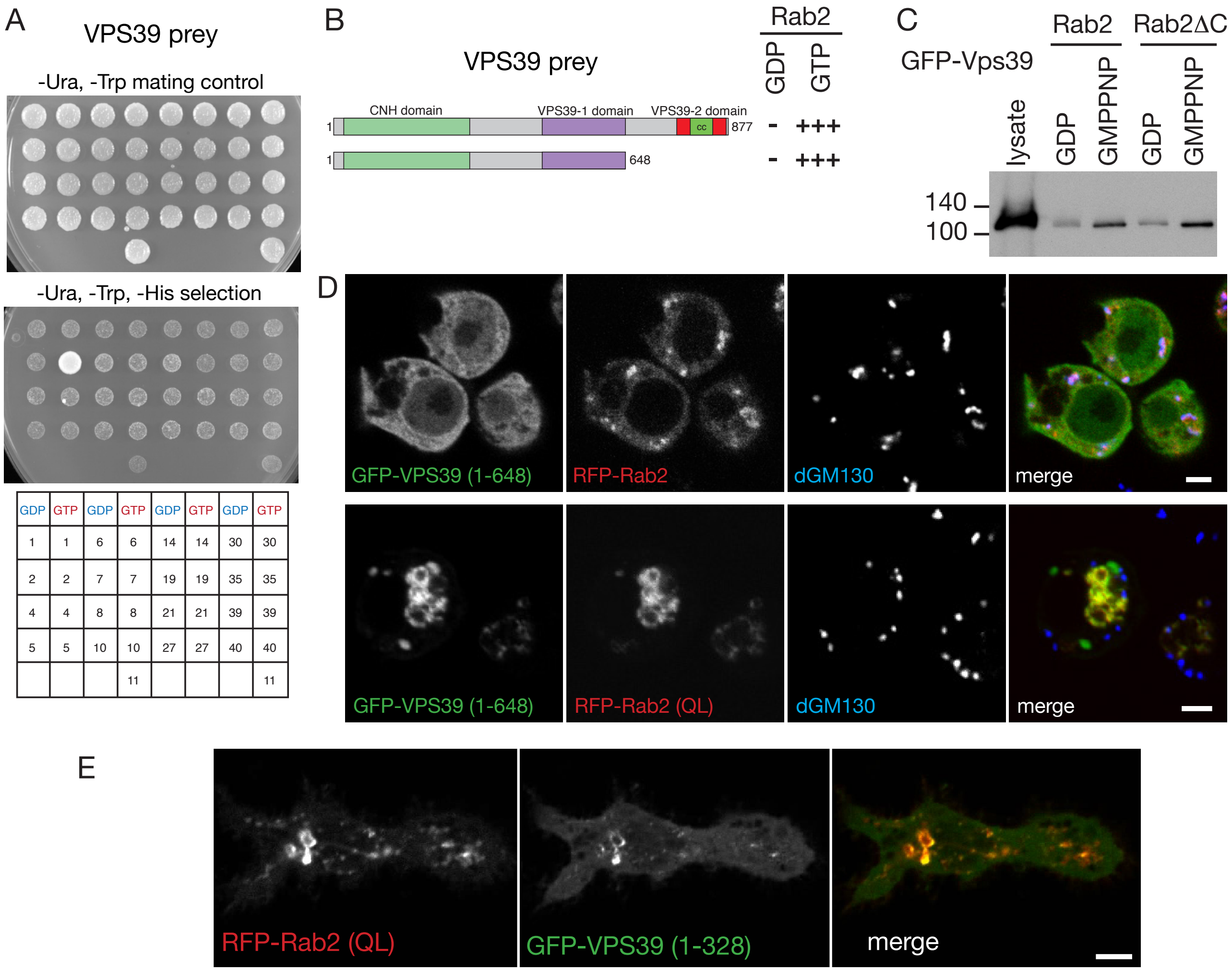
A
VPS39 prey
-Ura, -Trp mating control
-Ura, -Trp, -His selection
GDP GTP GDP GTP GDP GTP GDP GTP
1 1 6 6 14 14 30 30
2 2 7 7 19 19 35 35
4 4 8 8 21 21 39 39
5 5 10 10 27 27 40 40
11 11
B
VPS39 prey
Rab2
GDP GTP
CNH domain
VPS39-1 domain
VPS39-2 domain
cc
1
877
648
- +++
- +++
C
GFP-Vps39
Rab2
Rab2ΔC
lysate
GDP
GMPPNP
GDP
GMPPNP
140
100
D
GFP-VPS39 (1-648)
RFP-Rab2
dGM130
merge
GFP-VPS39 (1-648)
RFP-Rab2 (QL)
dGM130
merge
E
RFP-Rab2 (QL)
GFP-VPS39 (1-328)
merge

Figure S3

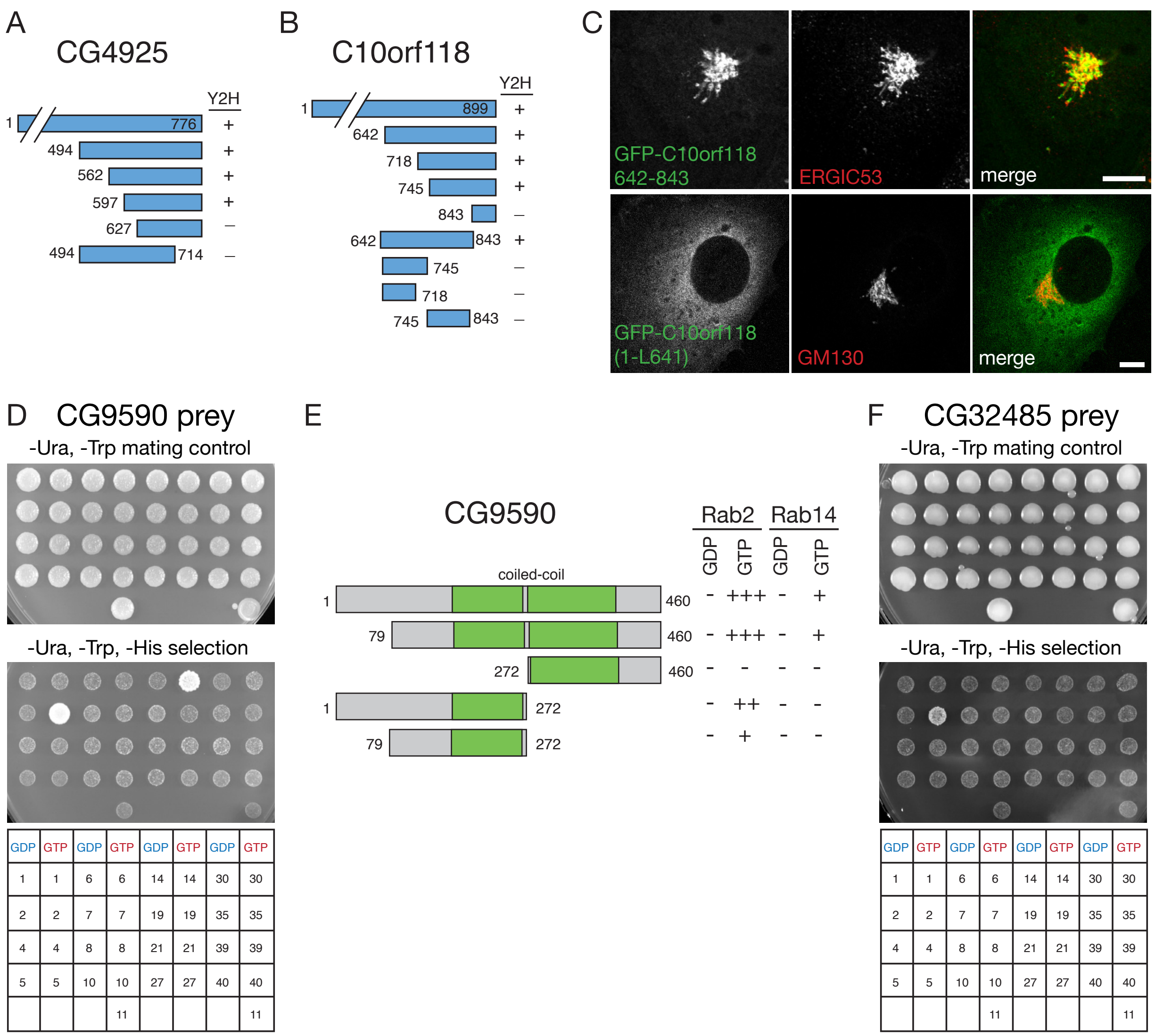
A
CG4925
Y2H
1
776
+
494
+
562
+
597
+
627
–
494
714
–
B
C10orf118
Y2H
1
899
+
642
+
718
+
745
+
843
–
642
843
+
745
–
718
–
745
843
–
C
GFP-C10orf118 642-843
ERGIC53
merge
GFP-C10orf118 (1-L641)
GM130
merge
D
CG9590 prey
-Ura, -Trp mating control
-Ura, -Trp, -His selection
GDP GTP GDP GTP GDP GTP GDP GTP
1 1 6 6 14 14 30 30
2 2 7 7 19 19 35 35
4 4 8 8 21 21 39 39
5 5 10 10 27 27 40 40
11 11
E
CG9590
coiled-coil
Rab2
Rab14
GDP GTP GDP GTP
1 460 - +++ - +
79 460 - +++ - +
272 460 - - - -
1 272 - ++ - -
79 272 - + - -
F
CG32485 prey
-Ura, -Trp mating control
-Ura, -Trp, -His selection
GDP GTP GDP GTP GDP GTP GDP GTP
1 1 6 6 14 14 30 30
2 2 7 7 19 19 35 35
4 4 8 8 21 21 39 39
5 5 10 10 27 27 40 40
11 11

Figure S4

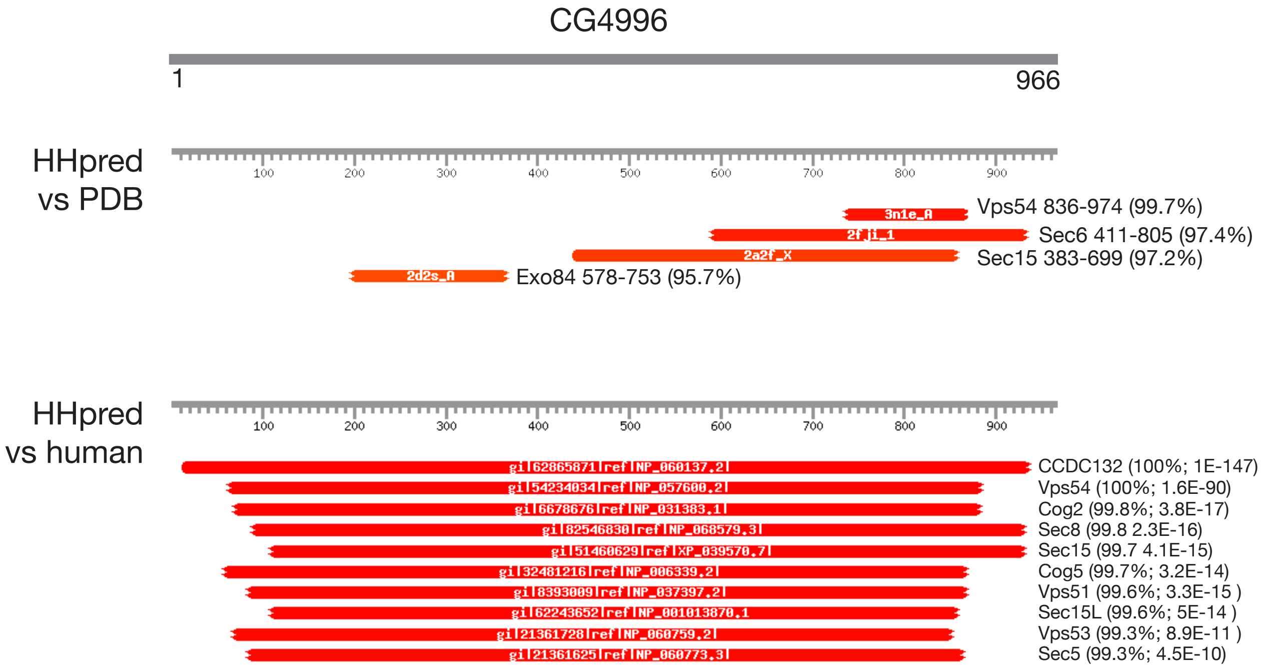
CG4996
1
966
HHpred vs PDB
100
200
300
400
500
600
700
800
900
3n1e_A
Vps54 836-974 (99.7%)
2fji_1
Sec6 411-805 (97.4%)
2a2f_X
Sec15 383-699 (97.2%)
2d2s_A
Exo84 578-753 (95.7%)
HHpred vs human
gi|62865871|ref|NP_060137.2|
CCDC132 (100%; 1E-147)
gi|54234034|ref|NP_057600.2|
Vps54 (100%; 1.6E-90)
gi|6678676|ref|NP_031383.1|
Cog2 (99.8%; 3.8E-17)
gi|82546830|ref|NP_068579.3|
Sec8 (99.8 2.3E-16)
gi|51460629|ref|XP_039570.7|
Sec15 (99.7 4.1E-15)
gi|32481216|ref|NP_006339.2|
Cog5 (99.7%; 3.2E-14)
gi|8393009|ref|NP_037397.2|
Vps51 (99.6%; 3.3E-15)
gi|62243652|ref|NP_001013870.1
Sec15L (99.6%; 5E-14)
gi|21361728|ref|NP_060759.2|
Vps53 (99.3%; 8.9E-11)
gi|21361625|ref|NP_060773.3|
Sec5 (99.3%; 4.5E-10)

Figure S5

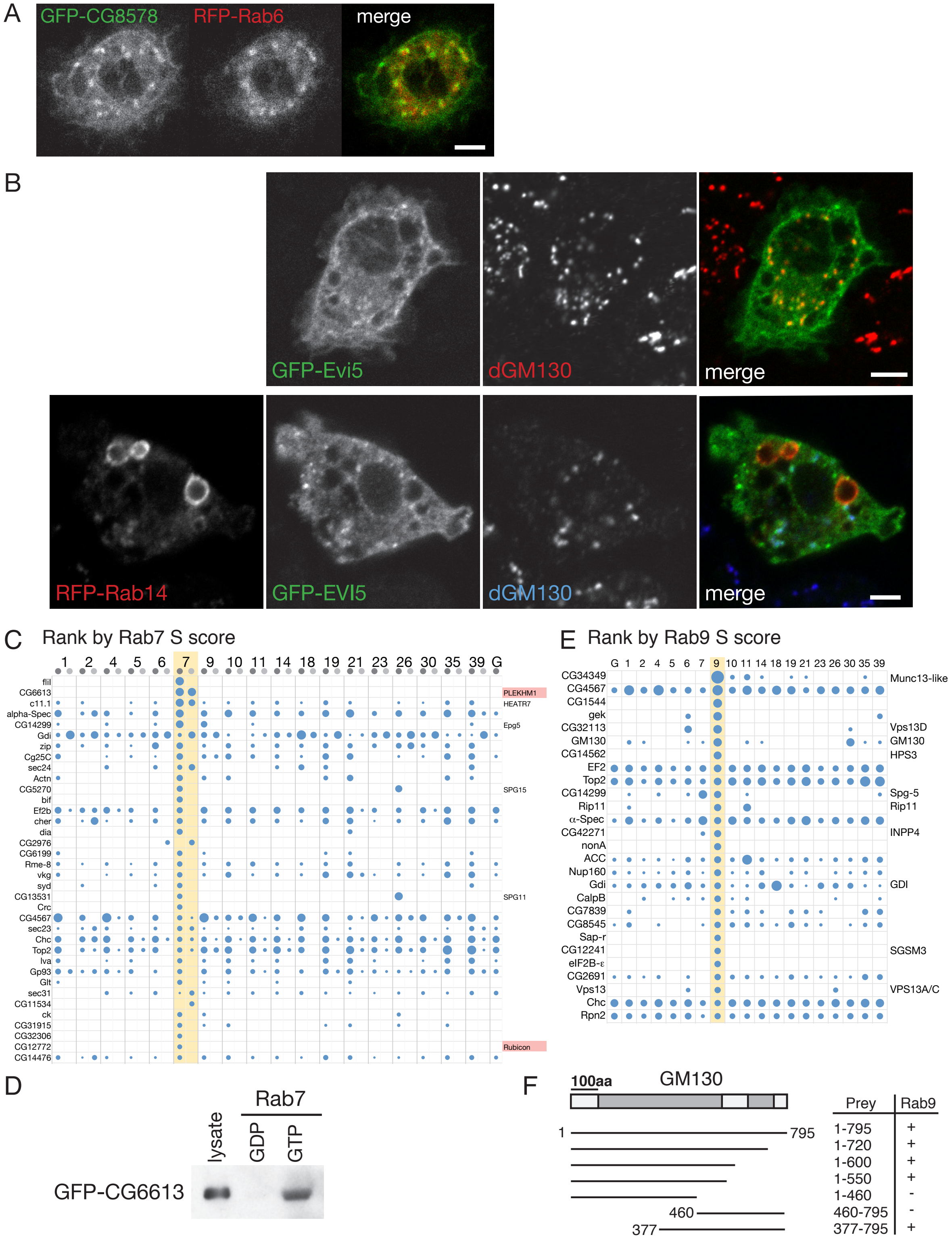
A
GFP-CG8578
RFP-Rab6
merge
B
GFP-Evi5
dGM130
merge
RFP-Rab14
GFP-EVI5
dGM130
merge
C
Rank by Rab7 S score
1 2 4 5 6 7 9 10 11 14 18 19 21 23 26 30 35 39 G
fliI
CG6613
c11.1
alpha-Spec
CG14299
Gdi
zip
Cg25C
sec24
Actn
CG5270
bif
Ef2b
cher
dia
CG2976
CG6199
Rme-8
vkg
syd
CG13531
Crc
CG4567
sec23
Chc
Top2
lva
Gp93
Glt
sec31
CG11534
ck
CG31915
CG32306
CG12772
CG14476
PLEKHM1
HEATR7
Epg5
SPG15
SPG11
Rubicon
D
Rab7
lysate
GDP
GTP
GFP-CG6613
E
Rank by Rab9 S score
G 1 2 4 5 6 7 9 10 11 14 18 19 21 23 26 30 35 39
CG34349
CG4567
CG1544
gek
CG32113
GM130
CG14562
EF2
Top2
CG14299
Rip11
α-Spec
CG42271
nonA
ACC
Nup160
Gdi
CalpB
CG7839
CG8545
Sap-r
CG12241
eIF2B-ε
CG2691
Vps13
Chc
Rpn2
Munc13-like
Vps13D
GM130
HPS3
Spg-5
Rip11
INPP4
GDI
SGSM3
VPS13A/C
F
100aa
GM130
1
795
460
377
Prey
Rab9
1-795 +
1-720 +
1-600 +
1-550 +
1-460 -
460-795 -
377-795 +

Figure S6

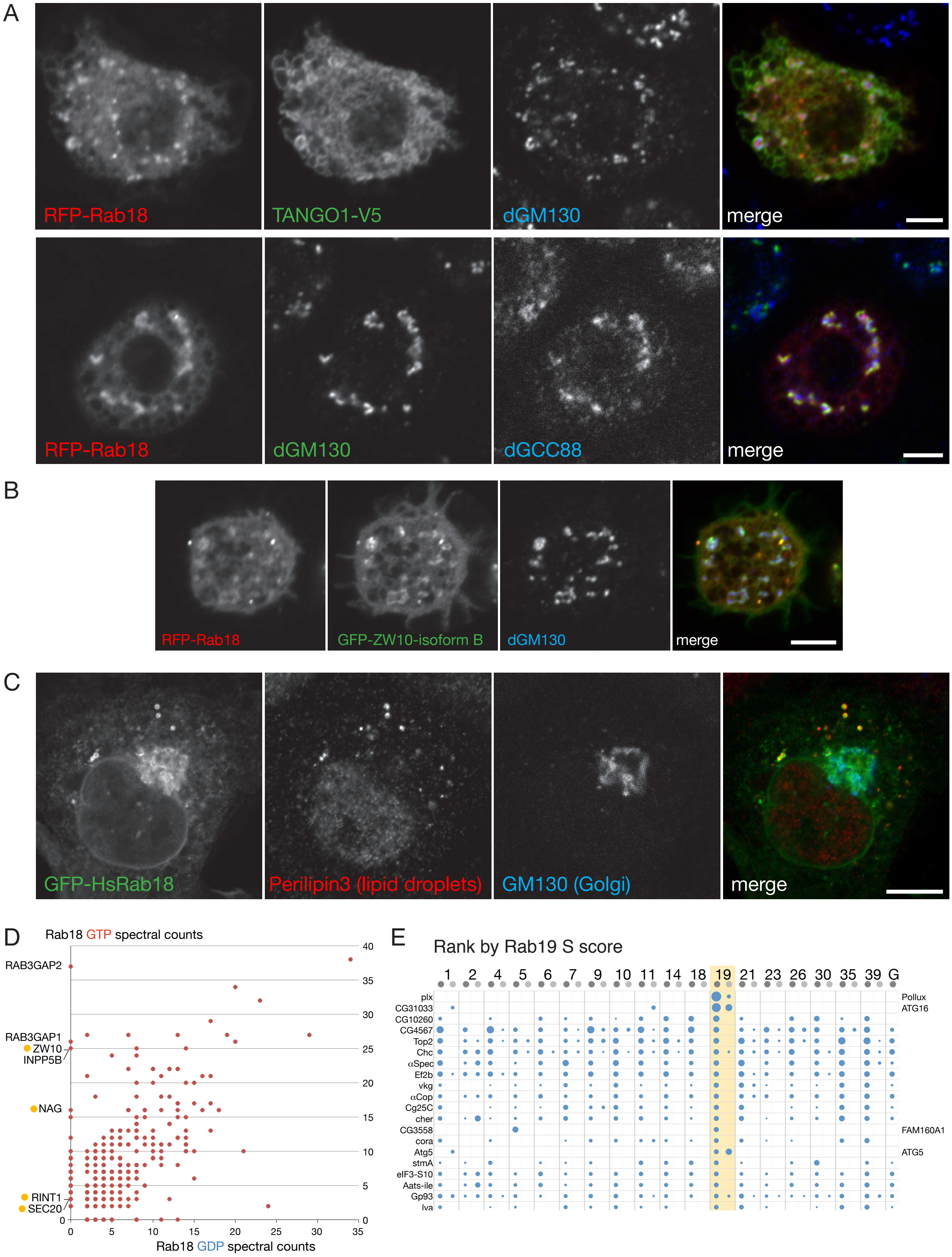

A
RFP-Rab18
TANGO1-V5
dGM130
merge
RFP-Rab18
dGM130
dGCC88
merge
B
RFP-Rab18
GFP-ZW10-isoform B
dGM130
merge
C
GFP-HsRab18
Perilipin3 (lipid droplets)
GM130 (Golgi)
merge
D
Rab18 GTP spectral counts
RAB3GAP2
RAB3GAP1
ZW10
INPP5B
NAG
RINT1
SEC20
0 5 10 15 20 25 30 35 40
0 5 10 15 20 25 30 35
Rab18 GDP spectral counts
E
Rank by Rab19 S score
1 2 4 5 6 7 9 10 11 14 18 19 21 23 26 30 35 39 G
plx
CG31033
CG10260
CG4567
Top2
Chc
αSpec
Ef2b
vkg
αCop
Cg25C
cher
CG3558
cora
Atg5
stmA
eIF3-S10
Aats-ile
Gp93
lva
Pollux
ATG16
FAM160A1
ATG5

Figure S7

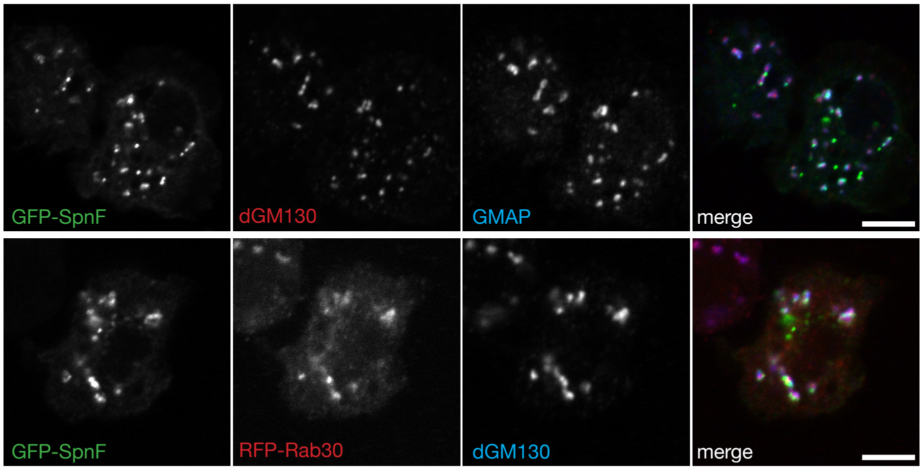

Supplemental Figure Legends

Figure S1. The Rab family of *Drosophila melanogaster*, related to Figure 1.

(A) Evolutionary relationship of all human and *Drosophila* Rabs compared to those of the last eukaryotic common ancestor (LECA) and a presumptive basal metazoan. The Rabs are shown in the families that expanded from the LECA (Kloepper et al., 2012). Grey shading indicates Rabs lost during metazoan evolution in either flies or humans. The four RabX proteins are those present in *Drosophila* but not humans (RabX2 and RabX3 are pseudogenes, and were not included in our study (Jin et al., 2012)), although recent phylogenetic analysis has suggested RabX5 may be the ortholog of human Rab34 (Elias et al., 2012; Kloepper et al., 2012). (B) mRNA levels of *Drosophila* Rabs in S2 cells (data from modENCODE (Contrino et al., 2012)). Those reported to be brain specific are in red (Chan et al., 2011; Jin et al., 2012). (C) Affinity chromatography of S2 cell lysate with GTP-locked Rabs 1, 2, 3, 4 and 5. Gels were transferred to nitrocellulose and immunoblotted for Hook (Krämer and Phistry, 1999).

Figure S2. Mapping the Rab2 binding site on VPS39, related to Figure 2.

(A) A truncated form of VPS39, VPS39(1-648aa) was fused to the Gal4 activation domain and tested against the indicated Rab proteins, either GDP or GTP-locked, by yeast two-hybrid assay. The upper panel shows the mating control, and the lower panel shows growth under selection for expression of the *HIS3* reporter gene. (B) Schematic diagram summarizing the interaction of VPS39 with Rab2 detected by yeast two-hybrid assay. (C) Affinity chromatography of lysates from S2 cells expressing GFP-VPS39 using a GST fusion to wild-type Rab2 loaded with GDP or the non-hydrolyzable GTP analog GMPPNP. Two forms of Rab2 were used with or without the C-terminal cysteines as indicated. Blots were probed with anti-GFP antibodies, and a consistent preference for the GTP form observed. (D) Confocal micrographs of *Drosophila* S2 cells co-expressing GFP-VPS39 (1-648aa) and either RFP-Rab2 or the GTP-locked mutant RFP-Rab2 (QL). Cells were labeled with antibodies against the Golgi marker dGM130. (E) A living cell expressing GFP-VPS39 (1-328) and the GTP-locked version of RFP-Rab2. Such co-localization was only seen at low expression levels. Scale bars 5 μm.

Figure S3. Rab2 interactions with two novel Golgi proteins, related to Figure 3.
(A) and (B) are summaries of data obtained from a series of yeast two-hybrid experiments with truncations of CG4925 and C10orf118 as depicted in the schematic diagrams. (C) Confocal micrographs of COS cells expressing GFP fusions to either the C-terminus of C10orf118 (residues 642-843, upper panel) or a form lacking the C-terminus (residues 1-643, lower panel). Cells were co-stained with antibodies against the Golgi markers ERGIC53 and GM130. Scale bars 10 μm. (D) Yeast two-hybrid assay using GDP and GTP-locked versions of a panel of Rab G-proteins as bait, and CG9590 fused to the C-terminus of the GAL4 activation domain as prey. The panels show the mating controls and growth under selection for expression of the *HIS3* reporter gene. (E) Schematic of the results of yeast 2-hybrid assays with truncations of CG9590 and nucleotide locked versions of Rab2 and Rab14. The green shading represents areas of coiled-coil in the CG9590 polypeptide. (F) As (D) but applied to CG32485.

Figure S4. CG4996 is a distant relative of Vps54, related to Figure 4.
(A) The results from a search for relatives of CG4996 in the Protein Data Bank (PDB) structures database (upper panels) or the human proteome (lower panels), using the structural-based homology detection program HHpred at default settings (Hildebrand et al., 2009). For PDB the top 4 hits are shown (all those where P>85%), and for the human proteins the top 10 hits (all those where P>99.25%), after duplicates were removed. The results reveal a very strong relationship to members of the CATCHR/quatrefoil family of tethering complexes that includes GARP, COG and the exocyst, with the highest score being with Vps54.

Figure S5. Effector proteins of Rab6, Rab7 and Rab9, related to Figure 5.
(A) Wide-field micrograph of a live cell co-expressing GFP-CG8578 and RFP-Rab6. (B) Confocal micrographs of cells expressing GFP-Evi5 alone (upper panels) or GFP-Evi5 and RFP-Rab14 (lower panels). Both co-stained for the Golgi marker dGM130. Co-expression of Rab14 enhances the Golgi localization of Evi5 but does not recruit the protein to endosomal ‘rings’. (C) Proteins isolated from cell lysates ranked by S-score for interaction with GST-Rab7 (detergent-free, lighter grey). The top 36 hits are shown from the full list in Table S3.

Known Rab7 effectors are marked in red, and other proteins with links to membrane traffic are indicated. (D) Affinity chromatography of S2 cell lysates expressing GFP-tagged CG6613 (the *Drosophila* ortholog of PLEKHM1) using Rab7 GDP- and GTP-locked mutants. Lysate represents 10% of the input. Blots were probed with antibodies against GFP. (E) Proteins isolated from cell lysates prepared with detergent lysis ranked by S-score for interaction with GST-Rab9. The top 27 hits are shown from the full list in Table S3. Proteins with links to membrane traffic are indicated. (F) dGM130 truncations used to map the Rab9 binding site by yeast two-hybrid assay. Interactions were detected by growth under *HIS3* selection. Scale bars 5 µm.

Figure S6. Rab18 has roles on the ER and the Golgi, related to Figure 6.
(A) Confocal micrographs of S2 cells expressing RFP-Rab18, and probed with antibodies against exogenous TANGO1-V5 (ER exit sites), or endogenous dGM130 (*cis*-Golgi) and dGCC88 (*trans*-Golgi). (B) Confocal micrograph of an S2 cell co-expressing RFP-Rab18 and GFP-ZW10 (isoform ZW10-PB), and probed with anti-dGM130 antibodies. (C) Confocal micrographs of COS cells expressing human Rab18 (GFP-HsRab18), and stained with antibodies against endogenous perilipin 3 and GM130 to label lipid droplets and the Golgi respectively. The GFP-HsRab18 is found on both structures, with such lipid droplet accumulation only seen in those cells with higher expression levels of GFP-HsRab18. (D) Comparison of the spectral counts for proteins isolated from human HEK293 cell lysates by affinity chromatography with GST tagged forms of GTP-locked or GDP-locked human Rab18. GTP-specific interactors with orthologs found in the *Drosophila* Rab18 dataset are labeled, ochre dots indicating NRZ subunits. For clarity the ten highest scoring interactors that were abundant in both datasets are not shown. (E) Proteins isolated from cell lysates ranked by S-score for interaction with GST-Rab19 (detergent-free, lighter grey). The top 20 hits are shown from the full list in Table S3. Specific interactors with possible links to membrane traffic are indicated. Scale bars 5 µm.

Figure S7. Ik2 binding partner SpnF is localized to the Golgi apparatus, related to Figure 7.

Confocal images of S2 cells expressing GFP-SpnF alone (upper panels), or with RFP-Rab30 (lower panels). Cells were fixed and stained with antibodies against the Golgi markers dGM130 and GMAP. Scale bars 5 μm.

Supplemental Tables

Table S1. Mass spectrometric analysis of proteins bound to Rabs in the detergent dataset, related to Figure 1.

Proteins identified as associating with at least one Rab in the dataset from lysates prepared with the detergent CHAPS. The spectral counts are in Sheet S1A, and the S scores in Sheet S1B (see tabs at bottom of sheet). For each protein the gene number, FlyBase gene number (FBgn), gene symbol, molecular weight and relative mRNA levels in S2 cells are stated. Provided as Excel file.

Table S2. Mass spectrometric analysis of proteins bound to Rabs in the detergent-free dataset, related to Figure 1.

Proteins identified as associating with at least one Rab in the dataset from lysates prepared without detergent. The spectral counts are in Sheet S2A, and the S scores in Sheet S2B (see tabs at bottom of sheet). For each protein the gene number, FlyBase gene number (FBgn), gene symbol, molecular weight and relative mRNA levels in S2 cells are stated. Provided as Excel file.

Table S3. Merged mass-spectrometric analysis from both datasets, related to Figures 2, 4-8.

Proteins identified as associating with at least one Rab in either dataset. The spectral counts are in Sheet S3A, and the S scores in Sheet S3B (see tabs at bottom of sheet). For each protein the gene number, gene symbol, human orthologs and molecular weight are stated. Provided as Excel file.

Extended Experimental Procedures

Plasmids and yeast two-hybrid assays

Drosophila and human Rab proteins lacking the C-terminal cysteine residues were cloned into pGEX6p1 for bacterial expression and into the bait vector pGBDU-C1 for yeast two-hybrid assays. Point mutations were introduced to create a panel of GDP-locked Rab proteins (either S->N or T->N as appropriate) or GTP-locked Rab proteins (Q->L, or A->L for dRab18) using the Quikchange mutagenesis kit according to the manufacturers instructions (Agilent Technologies). Effector proteins were amplified from EST clones and inserted into the prey vector pGAD424. Yeast two-hybrid assays were performed as previously described (James et al., 1996). For expression of tagged proteins in *Drosophila* tissue culture cells GFP, RFP and myc N-terminal tagged plasmids were constructed using either the Gateway vectors pAGW, pARW, or pAMW respectively (Drosophila Genomics Resource Center (DGRC)), or a modified version of pAC5.1/V5-HisA in which an N-terminal GFP or TagRFP cassette was inserted between KpnI and NotI and a GAGA linker prior to the insert. A stop codon was added after the open reading frame prior to the V5 tag. For C-terminal myc tagging the Gateway vector pAWM was used. Human genes *VPS51*, *C10orf118*, *FAM114A1* or *VPS53* were PCR amplified from EST clones and inserted into COS cell vectors containing the cytomegalovirus promoter and GFP or epitope tags. All PCR products were checked by sequencing. Other plasmids encoded *Drosophila* LRRK-myc (Dodson et al., 2012), or GFP-HsRab4A or GFP-Hs.Rab18 (Yoshimura et al., 2007).

Affinity chromatography

For affinity chromatography experiments GST-Rab fusion proteins were expressed in the *E. coli* strain BL21-GOLD (DE3; Agilent Technologies). Bacteria were grown at 37°C to an OD_{600} of 0.7 and induced with 100 μM IPTG overnight at 16°C. Cells were centrifuged and dounce homogenized and sonicated in lysis buffer (20 mM Tris-HCl, pH 8.0, 110 mM KCl, 5 mM $MgCl_2$, 1% CHAPs (omitted for non-detergent samples), 5 mM-β-mercaptoethanol, protease inhibitors and 200 μM GDP or the non-hydrolysable GTP analogue GppNHp (Sigma). The lysates were clarified by centrifugation at 12,000 g for 15 min and applied at saturating levels to Glutathione-Sepharose beads (GE Healthcare) to bind the GST-Rab

proteins to the beads. The beads were washed 3 times with lysis buffer. Lysates from S2 cells (D.Mel-2, Life Technologies) contained 10-20 mg/ml protein and were prepared as follows: for large scale experiments 5 x 10^8 cells were used per Rab protein and lysed in 5 ml of lysis buffer. For small scale experiments one T75 flask of S2 cells (approx. 5 x 10^7 cells) was transfected with 30 μg of effector DNA and carrier DNA (ratio 50:50) and incubated at 25°C for 36-48 h. For LRRK-myc expression was induced by the addition of 0.5 mM copper sulfate for 24 h (Dodson et al., 2012). Cells were harvested by centrifugation, washed and then lysed. For CHAPS lysis cells were dounce homogenized in lysis buffer (20 mM Tris-HCl, pH8, 110 mM KCl, 5 mM $MgCl_2$, 1% CHAPS (VWR), 5 mM β-mercaptoethanol, protease inhibitors in the absence of nucleotide) at 4°C. For detergent-free lysis cells were resuspended in the same buffer with the CHAPS omitted, dounce homogenized, then passed through a 30 gauge needle. Lysates were clarified by centrifugation at 50,000 g for 30 min at 4°C. For large scale experiments supernatants were incubated with 150 μl GST-Rab coated beads in the presence of 100 μM GppNHp for 2 h at 4°C. For small-scale experiments 50 μl GST-Rab coated beads were used and 100 μM GDP or GppNHp added. For human Rab18, Q67L (GTP) and N122I (GDP) versions lacking the last 8 C-terminal amino acids were expressed as GST fusion from pGEX6p2. Extracts were made from HEK293T cells and subjected to affinity chromatography essentially as for the S2 cells.

After binding beads were washed rapidly in lysis buffer at 4°C using Mobicol columns (2B Scientific). For samples prepared with detergent-free lysis buffer the final wash contained 0.5% Triton X-100 to reduce non-specific binding. For large-scale affinity chromatography the columns were eluted by the addition of a high salt buffer and the opposing nucleotide (20 mM Tris-HCl pH8, 1.5 M KCl, 20 mM EDTA, 5 mM β-mercaptoethanol, 5mM GDP or GppNHp) followed by chloroform/methanol protein precipitation. The samples were resuspended in 40 μl SDS-PAGE buffer for gel electrophoresis. Small-scale samples were eluted directly with 100 μl of SDS sample buffer. 10% of the eluate and 1% of the lysate was separated on SDS-PAGE gels, transferred to nitrocellulose and blotted with antibodies against either GFP (clones 7.1 and 13.1, Roche), BicD (1B11, Developmental Studies Hybridoma Bank (DSHB)), Ema (Kim et al., 2012), Rod and ZW-10 (Scaërou et al., 2001), dSec5 (Sommer et al., 2005), Ik2 (Oshima et al., 2006), dGM130 (ab30637, Abcam), GMAP (Friggi-Grelin, F. et al, 2006) or the myc tag (9E10, Sigma).

Mass spectrometry and data analysis

Samples obtained from affinity chromatography using lysates prepared with CHAPs were loaded on 4-20% Tris-glycine SDS-PAGE gels and run for a few centimetres. Proteins were stained with Coomassie brilliant blue. The entire gel lane between the stacking gel and the GST-Rab protein was excised and cut into three equal parts (and hence only proteins >45kDa were analysed). Data-dependent liquid chromatography MS/MS was performed by nanoflow reverse-phase liquid chromatography coupled to a mass spectrometer (Velos Orbitrap; Thermo, San Jose, CA).

The detergent-free samples were run on SDS-PAGE gels and the entire lane cut into 8-12 slices. Proteins within gel slices were first reduced and alkylated using dithiothreitol and iodoacetamide respectively and then digested to peptides using trypsin. Resultant peptides were eluted from the gel pieces in 15 μl of 0.1% formic acid. 5 μl of this was injected onto a reverse phase column (15 cm, 75 μm internal diameter C18 PepMap column) attached to a 1200 liquid chromatography system (Agilent). The column eluate was sprayed into an LTQ linear ion trap mass spectrometer (Thermo), operated in triple play mode. Resulting data files from all gel fractions were combined and converted to .dta format using Bioworks version 3.2 (Thermo) and the .dta files merged to form .mgf files, using an in house script (Shell Script). Searches were against FlyBase *Drosophila* genome release 5.9 (totalling 21,064 proteins). Scaffold software (Proteome Software Inc) was used to analyse MS/MS-based peptide and protein identifications. For both datasets protein identifications were accepted if they could be established at >95.0% probability and contained at least two identified peptides. Peptide identifications were accepted if they could be established at >80.0% probability for the detergent-free samples and >20% probability for the 'CHAPs' samples. Using these criteria the predicted false discovery rate (FDR) was <1%.

Confidence scores

The S-score is an empirical value developed as part of the CompPASS platform. S-scores were calculated for each interactor using the CompPASS algorithm. Briefly:

$S_{i,j} = \sqrt{(k/\sum f_{i,j})} \times X_{i,j}$; $f = 1$ if $X_{i,j} > 0$

where i is the bait number, j is the interactor and thus $X_{i,j}$ is the total spectral counts (referred to in this paper as "spectral counts") of prey i with bait j, K is the total number of baits in the dataset, and *f* is the frequency that prey i interacts with the set of baits. S-scores were calculated independently for each dataset.

Cell culture, transfection and immunofluorescence.

Drosophila S2 cells were grown at 25°C in serum-free medium (Express Five, Invitrogen) containing penicillin, streptomycin and L-glutamine. COS cells were grown at 37°C in DMEM supplemented with penicillin, streptomycin and 10% FCS. S2 cells were transfected in 6-well plates with 1 μg Rab plasmid DNA, 1 μg effector plasmid DNA and 1 μg carrier DNA (pAW empty vector (DGRC)) using Fugene HD (Promega) according to the manufacturer's instructions. For plasmids encoding Rab6, CG4925 or CG8578 0.3 μg of DNA was used with 1.7 μg carrier DNA. COS cells were transfected with 0.5 μg of Rab and effector plasmids using Fugene 6 according to the manufacturer's instructions (Promega). In all cases, cells were fixed in 4% formaldehyde in PBS and permeabilized in 0.5% Triton-X100/PBS. Cells were blocked for one hour in PBS containing 20% FCS and 0.25% Tween-20 and probed with the antibodies in the same buffer. Polyclonal antibodies against dGM130 (ab30637, Abcam), GMAP (Friggi-Grelin et al., 2006), dGCC88 and dGolgin-245 (Sinka et al., 2008), Hrs (Lloyd et al., 2002), Hook (Krämer and Phistry, 1996), human C10orf118 (HPA018019, Sigma), human ERGIC53 (ALX-804-602, Alexis), human GM130 (560257, BD Biosciences), human EEA1(610457, BD Biosciences), human ZW10 (ab21582, Abcam), or human perilipin 3 (ab47639, Abcam); or monoclonal antibodies against Rab7 (Tanaka and Nakamura, 2008), V5 (R960-25, Life Technologies), or the myc tag (9E10, Sigma) were detected with species-specific Alexa-labeled secondary antibodies (Molecular Probes). The cells were mounted in Vectashield (Vector Laboratories) and imaged using an LSM 780 confocal (Zeiss). At least 100 transfected cells were examined for each experiment and only images representative of phenomena visible in at least 80% are shown. For each example of a Rab stimulating recruitment we performed a control with a different Rab to confirm that the effect was specific. For live cell imaging S2 cells were transferred into Lab-Tek chambers (VWR) pre-coated with poly-L-lysine. Cells were settled for 30-60 min at 25°C then washed briefly with PBS prior to imaging.

Supplemental References

Chan, C.-C., Scoggin, S., Wang, D., Cherry, S., Dembo, T., Greenberg, B., Jin, E.J., Kuey, C., Lopez, A., Mehta, S.Q., et al. (2011). Systematic discovery of Rab GTPases with synaptic functions in *Drosophila*. Curr. Biol. *21*, 1704–1715.

Contrino, S., Smith, R.N., Butano, D., Carr, A., Hu, F., Lyne, R., Rutherford, K., Kalderimis, A., Sullivan, J., Carbon, S., et al. (2012). modMine: flexible access to modENCODE data. Nucleic Acids Res. *40*, D1082–D1088.

Dodson, M.W., Zhang, T., Jiang, C., Chen, S., and Guo, M. (2012). Roles of the *Drosophila* LRRK2 homolog in Rab7-dependent lysosomal positioning. Hum. Mol. Genet. *21,* 1350–1363.

Elias, M., Brighouse, A., Gabernet-Castello, C., Field, M.C., and Dacks, J.B. (2012). Sculpting the endomembrane system in deep time: high resolution phylogenetics of Rab GTPases. J. Cell Sci. *125*, 2500–2508.

Friggi-Grelin, F., Rabouille, C., and Therond, P. (2006). The cis-Golgi *Drosophila* GMAP has a role in anterograde transport and Golgi organization in vivo, similar to its mammalian ortholog in tissue culture cells. Eur. J. Cell Biol. *85*, 1155–1166.

Hildebrand, A., Remmert, M., Biegert, A., and Söding, J. (2009). Fast and accurate automatic structure prediction with HHpred. Proteins *77 Suppl 9*, 128–132.

James, P., Halladay, J., and Craig, E.A. (1996). Genomic libraries and a host strain designed for highly efficient two-hybrid selection in yeast. Genetics *144*, 1425–1436.

Jin, E.J., Chan, C.-C., Agi, E., Cherry, S., Hanacik, E., Buszczak, M., and Hiesinger, P.R. (2012). Similarities of *Drosophila* Rab GTPases based on expression profiling: completion and analysis of the Rab-Gal4 kit. PLoS ONE *7*, e40912.

Kim, S., Naylor, S.A., and DiAntonio, A. (2012). *Drosophila* Golgi membrane protein Ema promotes autophagosomal growth and function. Proc. Natl. Acad. Sci. USA *109*, E1072–E1081.

Kloepper, T., Kienle, N., Fasshauer, D., and Munro, S. (2012). Untangling the evolution of Rab G proteins: implications of a comprehensive genomic analysis. BMC Biol. *10*, 71.

Krämer, H., and Phistry, M. (1996). Mutations in the *Drosophila* hook gene inhibit endocytosis of the boss transmembrane ligand into multivesicular bodies. J. Cell Biol. *133*, 1205–1215.

Krämer, H., and Phistry, M. (1999). Genetic analysis of hook, a gene required for endocytic trafficking in *Drosophila*. Genetics *151*, 675–684.

Lloyd, T.E., Atkinson, R., Wu, M.N., Zhou, Y., Pennetta, G., and Bellen, H.J. (2002). Hrs regulates endosome membrane invagination and tyrosine kinase receptor signaling in

Drosophila. Cell *108*, 261–269.

Oshima, K., Takeda, M., Kuranaga, E., Ueda, R., Aigaki, T., Miura, M., and Hayashi, S. (2006). IKK epsilon regulates F actin assembly and interacts with *Drosophila* IAP1 in cellular morphogenesis. Curr. Biol. *16*, 1531–1537.

Scaërou, F., Starr, D.A., Piano, F., Papoulas, O., Karess, R.E., and Goldberg, M.L. (2001). The ZW10 and Rough Deal checkpoint proteins function together in a large, evolutionarily conserved complex targeted to the kinetochore. J. Cell Sci. *114*, 3103–3114.

Sinka, R., Gillingham, A.K., Kondylis, V., and Munro, S. (2008). Golgi coiled-coil proteins contain multiple binding sites for Rab family G proteins. J. Cell Biol. *183*, 607–615.

Sommer, B., Oprins, A., Rabouille, C., and Munro, S. (2005). The exocyst component Sec5 is present on endocytic vesicles in the oocyte of *Drosophila melanogaster*. J. Cell Biol. *169*, 953–963.

Sowa, M.E., Bennett, E.J., Gygi, S.P., and Harper, J.W. (2009). Defining the human deubiquitinating enzyme interaction landscape. Cell *138*, 389–403.

Tanaka, T., and Nakamura, A. (2008). The endocytic pathway acts downstream of Oskar in *Drosophila* germ plasm assembly. Development *135*, 1107–1117.

Yoshimura, S.-I., Egerer, J., Fuchs, E., Haas, A.K., and Barr, F.A. (2007). Functional dissection of Rab GTPases involved in primary cilium formation. J. Cell Biol. *178*, 363–369.
